# Assessing tumor microstructure with time‐dependent diffusion imaging: Considerations and feasibility on clinical MRI and MRI‐Linac

**DOI:** 10.1002/mp.17453

**Published:** 2024-10-10

**Authors:** Minea Jokivuolle, Faisal Mahmood, Kristoffer Hougaard Madsen, Frederik Severin Gråe Harbo, Lars Johnsen, Henrik Lundell

**Affiliations:** ^1^ Laboratory of Radiation Physics Department of Oncology Odense University Hospital Odense Denmark; ^2^ Department of Clinical Research University of Southern Denmark Odense Denmark; ^3^ Danish Research Centre for Magnetic Resonance Centre for Functional and Diagnostic Imaging and Research Copenhagen University Hospital ‐ Amager and Hvidovre Hvidovre Denmark; ^4^ Department of Applied Mathematics and Computer Science Technical University of Denmark Kongens Lyngby Denmark; ^5^ Department of Radiology Odense University Hospital Odense Denmark; ^6^ Department of Health Technology Technical University of Denmark Kongens Lyngby Denmark

**Keywords:** biologically guided radiotherapy, biophysical modeling, diffusion MRI, imaging biomarkers, time‐dependent diffusion

## Abstract

**Background:**

Quantitative imaging biomarkers (QIBs) can characterize tumor heterogeneity and provide information for biological guidance in radiotherapy (RT). Time‐dependent diffusion MRI (TDD‐MRI) derived parameters are promising QIBs, as they describe tissue microstructure with more specificity than traditional diffusion‐weighted MRI (DW‐MRI). Specifically, TDD‐MRI can provide information about both restricted diffusion and diffusional exchange, which are the two time‐dependent effects affecting diffusion in tissue, and relevant in tumors. However, exhaustive modeling of both effects can require long acquisitions and complex model fitting. Furthermore, several introduced TDD‐MRI measurements can require high gradient strengths and/or complex gradient waveforms that are possibly not available in RT settings.

**Purpose:**

In this study, we investigated the feasibility of a simple analysis framework for the detection of restricted diffusion and diffusional exchange effects in the TDD‐MRI signal. To promote the clinical applicability, we use standard gradient waveforms on a conventional 1.5 T MRI system with moderate gradient strength (*G*_max_ = 45 mT/m), and on a hybrid 1.5 T MRI‐Linac system with low gradient strength (*G*_max_ = 15 mT/m).

**Methods:**

Restricted diffusion and diffusional exchange were simulated in geometries mimicking tumor microstructure to investigate the DW‐MRI signal behavior and to determine optimal experimental parameters. TDD‐MRI was implemented using pulsed field gradient spin echo with the optimized parameters on a conventional MRI system and a MRI‐Linac. Experiments in green asparagus and 10 patients with brain lesions were performed to evaluate the time‐dependent diffusion (TDD) contrast in the source DW‐images.

**Results:**

Simulations demonstrated how the TDD contrast was able to differentiate only dominating diffusional exchange in smaller cells from dominating restricted diffusion in larger cells. The maximal TDD contrast in simulations with typical cancer cell sizes and in asparagus measurements exceeded 5% on the conventional MRI but remained below 5% on the MRI‐Linac. In particular, the simulated TDD contrast in typical cancer cell sizes (*r* = 5–10 µm) remained below or around 2% with the MRI‐Linac gradient strength. In patients measured with the conventional MRI, we found sub‐regions reflecting either dominating restricted diffusion or dominating diffusional exchange in and around brain lesions compared to the noisy appearing white matter.

**Conclusions:**

On the conventional MRI system, the TDD contrast maps showed consistent tumor sub‐regions indicating different dominating TDD effects, potentially providing information on the spatial tumor heterogeneity. On the MRI‐Linac, the available TDD contrast measured in asparagus showed the same trends as with the conventional MRI but remained close to typical measurement noise levels when simulated in common cancer cell sizes. On conventional MRI systems with moderate gradient strengths, the TDD contrast could potentially be used as a tool to identify which time‐dependent effects to include when choosing a biophysical model for more specific tumor characterization.

## 1 INTRODUCTION

In standard radiotherapy (RT), a uniform dose distribution is prescribed to the gross tumor volume (GTV) irrespective of spatial tumor heterogeneity.^1^, ^2^ The heterogeneity may account for poor local tumor control if not taken into account.^3^, ^4^ For example, sub‐regions with higher cell density may require higher doses to achieve local control.^3^ On the other hand, lowering doses to regions with less viable cells may help to reduce damage to the surrounding healthy tissue. Accounting for the tumor heterogeneity requires the development of quantitative imaging biomarkers (QIBs) capable of assessing the heterogeneity.^1^, ^2^, ^4^, ^5^

Diffusion‐weighted magnetic resonance imaging (DW‐MRI) sensitizes the magnetic resonance (MR) signal to the diffusive motion of water molecules, which makes it a powerful tool to probe tissue microstructure and potential tumor heterogeneity.^5^, ^6^ The apparent diffusion coefficient (ADC) derived from conventional DW‐MRI is widely investigated in RT as a potential QIB.^7^, ^8^, ^9^, ^10^ Low ADC has been linked to high cell density,^11^, ^12^ but contradicting results have also been reported,^12^ which potentially hinders the validation of ADC as a QIB and its clinical use. While ADC in general decreases as cell density increases, it may also depend on other factors such as cell size (restricted diffusion) and membrane permeability (diffusional exchange).^13^, ^14^

An advanced DW‐MRI method, time‐dependent diffusion MRI (TDD‐MRI), provides additional information about the diffusion process, enabling a more specific assessment of tissue microstructure.^15^, ^16^ By fitting a suitable mathematical model to a TDD‐MRI measurement, it is possible to infer estimates of several aspects of tissue microstructure.^15^, ^17^, ^18^, ^19^, ^20^ Using such models, several prior studies have investigated TDD‐MRI in tumors, and found correlation between TDD‐MRI parameters and tumor grade^21^, ^22^, ^23^, ^24^ or type,^25^ and verified TDD‐MRI estimates for cell density with histology.^22^, ^24^, ^26^

The challenge with existing TDD‐MRI works for cancer applications is that the principal models have focused on modeling either restricted diffusion^17^, ^18^, ^19^ or diffusional exchange^20^, ^25^ separately. Recent studies have demonstrated how exchange should be included in the biophysical models when characterizing tumors.^27^, ^28^ Although such results hold great potential in accurate tumor microstructure characterization, simultaneous estimation of both restricted diffusion and exchange can quickly increase the complexity of the model fitting, and can potentially require long measurement times to collect sufficient data. Furthermore, using models inherently carries the risk of estimating biased or uninformative parameters, if model assumptions are violated.

To avoid complex model fitting and prior assumptions of the tissue microstructure approaches focusing on the mere characterization of the time dependence of the diffusivity have been suggested and investigated in tumors^29^, ^30^, ^31^, ^32^, ^33^, ^34^, ^35^, ^36^ and in neuronal tissue.^37^, ^38^, ^39^ Examples include the *diffusion time‐dependent contrast* explored in the glioma model,^29^ and *temporal diffusion ratio* demonstrated in rat spinal cord.^39^ However, also here the existing works in tumors, and especially in neuronal tissue, have focused on restricted diffusion effects, neglecting diffusional exchange.

This feasibility study aims to add knowledge of both restricted diffusion and diffusional exchange effects in tumors using a simple analysis framework free of any prior assumptions of the tumor microstructure. To promote possible clinical applications in the future, we use clinical MRI scanners with limited gradient strengths (≤45 mT/m). We hypothesize that the generally larger sizes of tumor cells^15^, ^40^ compared to those of healthy neuronal cells^41^ may provide an unexploited potential of TDD‐MRI on MRI systems with limited gradient performance, compared to brain imaging, where strong gradients are required to achieve sensitivity to relevant microstructure, for example, the small axon diameters.^42^, ^43^ The hybrid MRI linear accelerator (MRI‐Linac), which allows online RT treatment adaptation, is an additional reason to investigate TDD‐MRI on systems with limited gradient strengths, as the current MRI‐Linacs have only modest gradient strengths (15–18 mT/m).^44^, ^45^

In this study, we explore the potential of TDD‐MRI for tumor characterization on a conventional 1.5 T MRI system and, for the first time, on a 1.5 T MRI‐Linac system. First, we provide a basic theoretical background to TDD‐MRI. We then present results from simulations in different tissue microstructures to evaluate the feasibility of TDD‐MRI in terms of detecting the effects of cell sizes (restricted diffusion) and membrane permeability (diffusional exchange), and for determining optimal experimental parameters. In line with earlier works at 3 T,^46^ we evaluate TDD‐MRI in a phantom with both Gaussian and non‐Gaussian diffusion compartments (water and green asparagus)^46^, ^47^, ^48^, ^49^ before proceeding to measurements in patients with brain lesions. We evaluate the time‐dependent diffusion (TDD) effects in terms of available contrasts in the source data (DW‐images) using a simple analysis framework, which can identify both dominating restriction and dominating exchange effects.

## 2 THEORY

### 2.1 Time‐dependent diffusion

**FIGURE 1:**
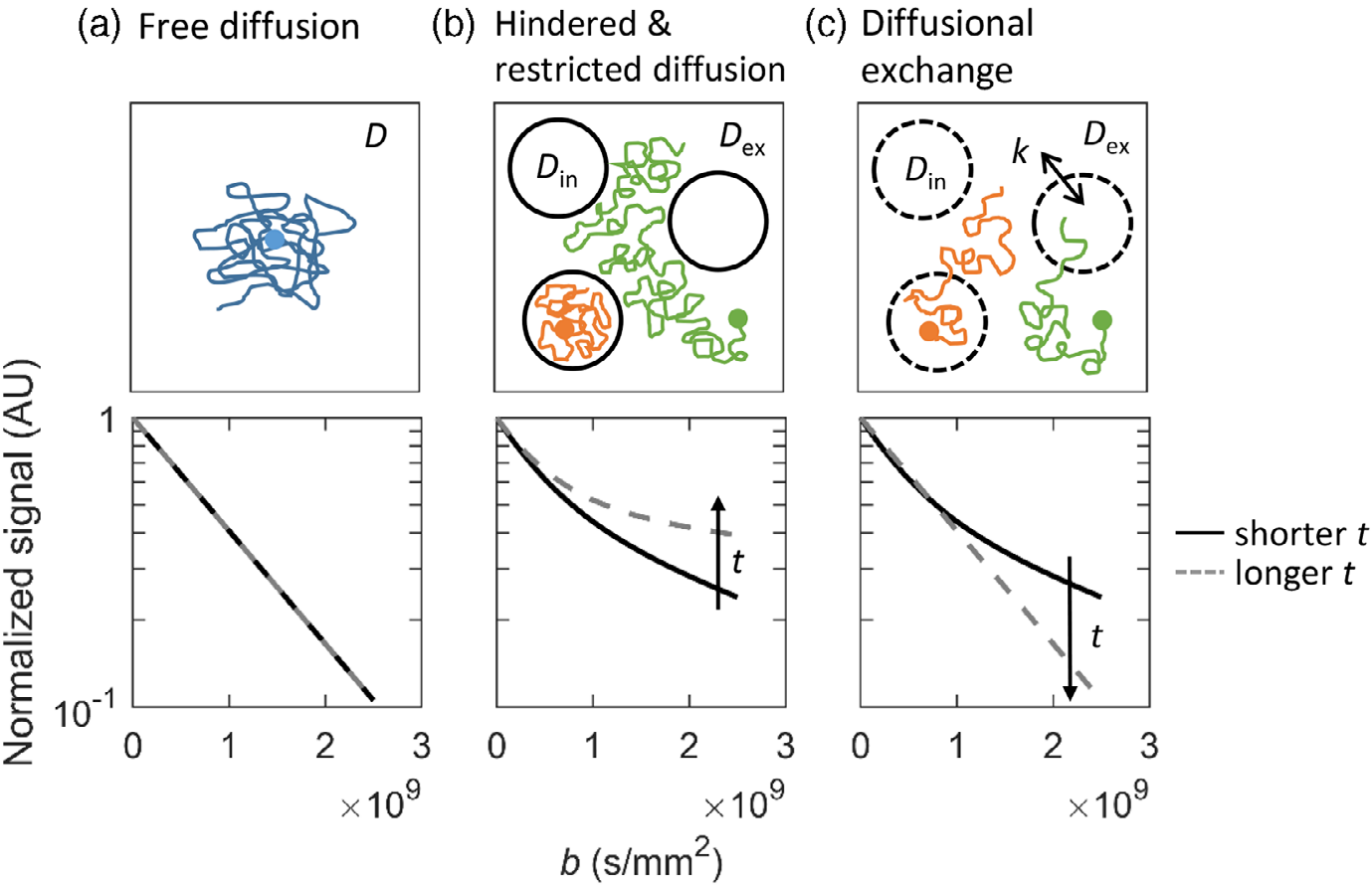
Illustrations of free diffusion (a), hindered and restricted diffusion (b), and diffusional exchange (c). Top row: Schematic representations of diffusion trajectories. Colored dots show the initial positions of example water molecules (blue: free diffusion; green: hindered diffusion; orange: restricted diffusion). Colored lines indicate the diffusion trajectories. Black circles indicate cell membranes (middle and right panel). *D* = diffusivity in free diffusion, *D*_in_ = intracellular diffusivity, *D*_ex_ = extracellular diffusivity, *k* = exchange rate. Bottom row: Representative DW‐MRI signal decay curves (AU) as a function of diffusion weighting (*b‐*value). Solid curve = DW‐MRI signal obtained with a shorter diffusion time (*t*). Dashed curve = signal obtained with a longer *t*. AU, arbitrary units; DW‐MRI, diffusion‐weighted magnetic resonance imaging.

In free diffusion (Figure 1a), the root mean square displacement of water molecules l(*t*) follows a Gaussian distribution according to the relation

(1) $$l\left(t\right)=\sqrt{2tD}.$$

Here, *t* is the diffusion time and D is diffusivity. In free diffusion, the diffusivity D equals the self‐diffusion coefficient of the medium, which is constant and independent of *t*. In tissue, the water molecules interact with tissue microstructure, such as cell membranes, which can make the diffusivity dependent on *t* and the distribution of l(*t*) non‐Gaussian.^50^ This time‐dependent or non‐Gaussian diffusion can be divided into hindered diffusion, restricted diffusion (Figure 1b), and diffusional exchange (Figure 1c). In hindered diffusion, which is often thought to take place in the extracellular compartment, the diffusivity D is reduced by obstacles but the l(*t*) is still unlimited making the hindered diffusion only weakly time‐dependent.^51^, ^52^ In restricted diffusion, the water molecules are trapped inside closed compartments, such as cells. With sufficient *t*, the l(*t*) is limited by the dimensions of the restriction and the diffusivity is reduced.^50^

Diffusional exchange, on the other hand, mixes water between different compartments, such as the intra‐ and extracellular spaces.^53^ Exchange adds a further time dependency to the diffusivity, as an increasing amount of intracellular water molecules venture into the extracellular space, allowing larger displacements over time. Contrary to the effect of restrictions, this increases the total diffusivity as a function of *t* with observation times matching the intracellular lifetime of the water.^15^, ^53^ Thus, diffusional exchange can confound the measurements of restricted diffusion and vice versa.

### 2.2 Measuring restricted diffusion and diffusional exchange with TDD‐MRI

**FIGURE 2:**
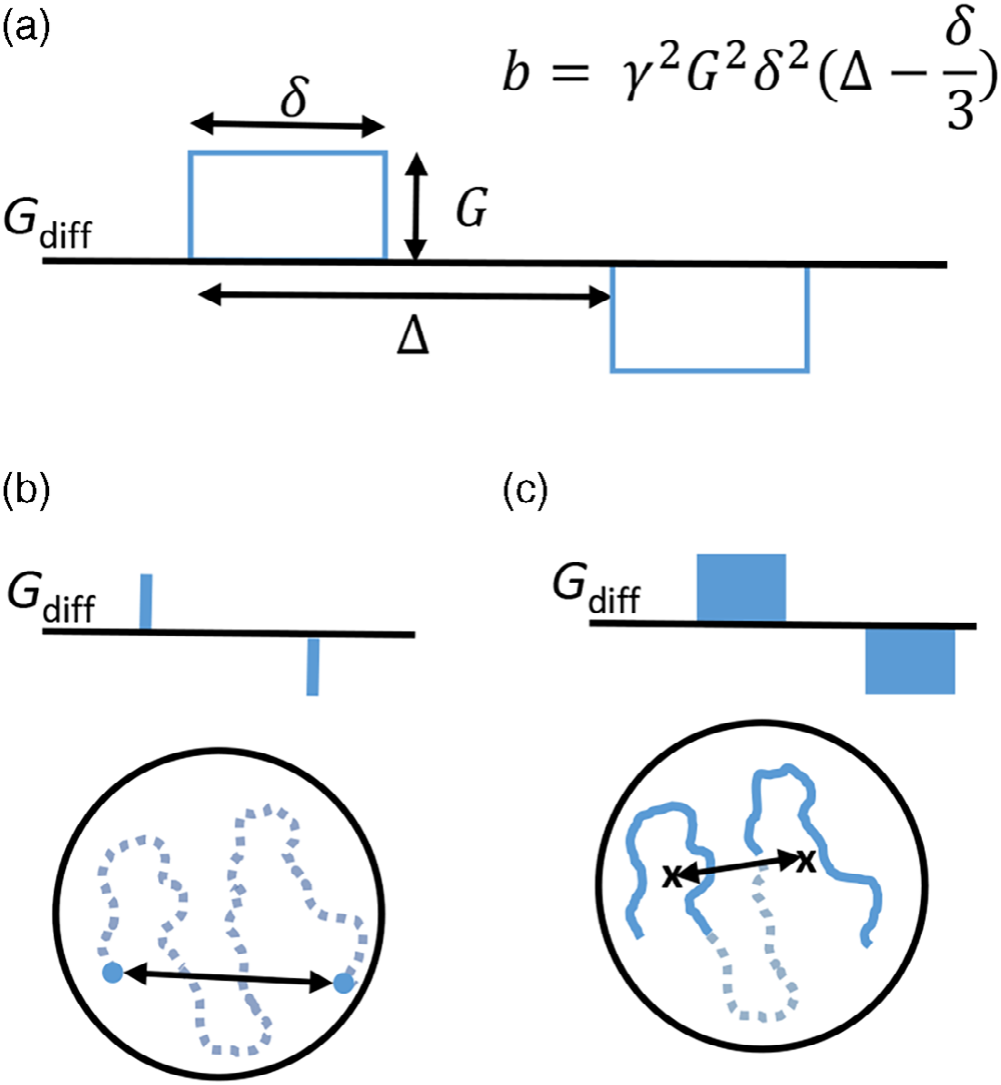
Measuring diffusion. (a) A schematic of the PGSE diffusion encoding gradients (*G*_diff_). The measurement parameters *δ* (gradient duration), *∆* (gradient separation), and *G* (gradient strength) together with the gyromagnetic ratio (*γ*) define the amount of diffusion weighting (*b*) for square wave gradients. (b) Measurable diffusion length (black arrow) within a closed compartment (black circle) with infinitely short gradient pulses, which fulfill the SGP condition. Blue dots indicate the diffusional motion during the gradient pulses, whereas the dashed line indicates the diffusional motion between the gradients. (c) Measurable diffusion length (black arrow) with realistic, long gradient pulses. The solid parts of the diffusion trajectory indicate motion during the diffusion gradients, and the center‐of‐masses of these two trajectories are marked with crosses. PGSE, pulsed field gradient spin echo; SGP, short gradient pulse.

The MR signal can be sensitized to diffusion with a pair of strong diffusion gradients that encode the positions of water molecules in the proton spin phase at two time points, as is done in the pulsed field gradient spin echo (PGSE) experiment.^54^ Diffusion spreads the water molecules and leads to a dispersion of spin phases that attenuates the total signal. Under the assumption of free diffusion, the attenuation is given by *S* = *S*_0_exp(‐*bD*), where *S_0_* is the MR signal without diffusion‐encoding gradients and *b* is the amount of diffusion weighting, which is controlled by the diffusion‐encoding gradient parameters (Figure 2a). From a repeated set of measurements with different diffusion weightings, it is possible to estimate the value of the diffusivity *D* as the slope of a mono‐exponential fit to the signal (Figure 1, bottom row). In the case of free diffusion, the measured *D* corresponds to the intrinsic diffusivity of water, whereas in tissue, it is replaced by the ADC reflecting the mean diffusivity within a voxel.

The effective diffusion time, *t_d_* $=\Delta -\frac{\delta }{3}$, defines the sensitivity of the TDD‐MRI measurement to the tissue microstructure in a PGSE experiment. Here, *δ* is the duration of one diffusion gradient lobe and *∆* is the leading edge separation of the gradient lobes. In order for the DW‐MRI signal decay to contain information about the tissue microstructure, *t_d_* needs to be sufficiently long. Based on Equation (1), usually *t_d_* > > $\frac{{l_{c}}^{2}}{D}$, where $l_{c}$ is the characteristic restriction length present in the tissue (e.g., cell radius).^55^

The total duration of *t_d_* is not the only relevant parameter when determining the measurement sensitivity to tissue microstructure, as the water molecules diffuse also during the diffusion gradients. Ideally, the diffusion encoding gradients would be infinitely short, making it possible to probe the full diffusion trajectories (l(*t_d_*)) (Figure 2b).^56^ This is generally referred to as the short gradient pulse (SGP) limit approximation.^57^ The SGP limit approximation can be used if *δ* < < $\frac{{l_{c}}^{2}}{D}$.^57^ In reality, this condition is rarely met, mainly due to limited gradient performance and the need for high *b*‐values. With longer *δ*, it is possible to probe the diffusive motion only between the *center of masses* of the diffusion trajectories taking place during the gradient pulses (Figure 2c).^56^ This makes the measured l(*t_d_*) appear shorter. When the diffusion length during the gradient pulse (l(*δ*)) equals the restriction size, the center‐of‐mass (COM) effect makes the probed restriction size approach zero.^56^

Although the COM effect sets a limit for the detectable restriction sizes, it can be exploited when trying to separate restricted diffusion effects in small cells from restriction in larger cells or diffusional exchange effects. Modulating *δ* on a range where the restriction sizes change from detectable to undetectable due to the COM effect can create a contrast in small restriction sizes. On the other hand, detection of diffusional exchange or larger restrictions requires longer *t_d_* and these are mainly detected with an overall change in *t_d_*, which is most efficiently achieved by modulating *∆* while keeping *δ* constant.

## 3 METHODS

### 3.1 Time‐dependent diffusion contrast

Due to TDD effects, that is, restricted diffusion and diffusional exchange, it is possible to contrast two DW‐MRI signals measured with different effective diffusion times but the same *b*‐value to obtain information about the tissue microstructure. We define the time‐dependent diffusion contrast (TDD contrast) for a specific *b*‐value as a subtraction of two normalized DW‐MRI signals:

(2) $$\mathrm{TDD}\;\mathrm{contrast}=\frac{S_{b}\left(t_{d,2}\right)}{S_{0}\left(t_{d,2}\right)\;}-\frac{S_{b}\left(t_{d,1}\right)}{S_{0}\left(t_{d,1}\right)},$$

where *S_b_* is the DW‐MRI signal with a non‐zero *b*‐value, *S*_0_ is the signal with *b* = 0 s/mm^2^, and *t_d_*_,1_ and *t_d_*_,2_ are the effective diffusion times used in the acquisition (*t_d_*_,2_ > *t_d_*_,1_). The sign and magnitude of the TDD contrast reflect the tissue microstructure affecting the diffusion and depend on the acquisition parameters, that is, the *b*‐value and *t_d_*. In general, positive TDD contrast indicates dominating restricted diffusion, while negative TDD contrast indicates dominating diffusional exchange. The TDD contrast is comparable across subjects due to the normalization and does not involve assumptions of model parameters or numerical fitting.

### 3.2 Simulations

The Camino Diffusion MRI toolkit (2.35.3, Microstructure Imaging Group, University College London, London, UK)^58^, ^59^ was used to perform Monte Carlo random‐walk simulations to explore the theoretically available TDD contrast over a large range of diffusion gradient waveforms in a few biologically relevant geometries mimicking tissue microstructure and with gradient strengths corresponding to clinical MRI systems (*G*_max_ = [15, 45] mT/m). Tissue microstructure was modeled with a three‐dimensional voxel consisting of square‐packed periodic spheres with 10 different radii (*r* = 2–20 µm) and intracellular volume fraction governing the cell density (*v*_in_ = 0.11, 0.30, 0.52). The permeability of the cell membranes was modeled with exchange rates *k* = 0, 1, 2, 8, 20 s^−1^, which were converted to probabilities for the Monte Carlo simulations.^60^ The intrinsic diffusivity was set to *D*_0_ = 2.0 µm^2^/ms. With each of the tissue microstructure configurations, the diffusion trajectories were generated from *t* = 0 to *t* = 250 ms with a constant time step *∆t* = 1e^−4^ ms and with 10^5^ diffusing particles randomly placed to both intra‐ and extracellular compartments. The diffusion‐weighted signals were calculated from the spin phase of the diffusing particles as accumulated by the gradient waveforms along one gradient axis. Both intra‐ and extracellular spins were included in the total diffusion signal. The simulations were used to determine optimized gradient waveforms (WFs) that maximized the TDD contrast.

The code for generating and visualizing the simulation results was written in Matlab (R2022a, MathWorks Inc., Natick, MA, USA). The simulation results are available at https://github.com/mjokivuolle/Jokivuolle_MedPhys_2024 with a Matlab script for visualization.

### 3.3 Phantom preparation and optical microscopy

A phantom for measuring both Gaussian and non‐Gaussian diffusion was used to validate the TDD‐MRI acquisition before proceeding to measurements in patients. The phantom was constructed by submerging stems of fresh green asparagus (*Asparagus officinalis*), bought from a local grocery store, in tap water. The water surrounding the asparagus stems provided a compartment for measuring Gaussian diffusion, whereas the asparagus stems provided a compartment for measuring non‐Gaussian diffusion, as previously demonstrated in the literature.^46^, ^47^, ^48^, ^49^ The TDD‐MRI measurements were performed after the temperature of the phantom had stabilized at room temperature. To obtain histological information of the asparagus microstructure, transversal sections of seven asparagus stems were stained with hematoxylin and eosin and digitized (NanoZoomer 2.0‐HT, Hamamatsu Photonics, Hamamatsu City, Japan). Digitized images were visualized in NDP Viewer (2.9.29, Hamamatsu Photonics, Hamamatsu City, Japan) and two 300 × 300 µm regions of interest (ROIs) were drawn in each section: one in the central and one in the periphery region. Average cell size in both regions was obtained by measuring the short and long axes of 20 cells in each ROI and by averaging the results across the seven stems.

### 3.4 Patients

Patients with brain lesions referred to treatment at Odense University Hospital (Odense, Denmark), were recruited for TDD‐MRI scanning after providing informed consent. The inclusion criteria were: patient was referred to treatment for brain lesion(s) at Odense University Hospital, age ≥ 18 years, and was able to understand written and verbal information given about the study. The exclusion criteria were: contraindications to MRI, metal in the imaging volume, cannot give informed consent, and chronic back pain making it difficult to lie still on MRI table. The study on patients was conducted in accordance with the Declaration of Helsinki, and approved by the local Institutional Review Board (case no. 23/48724).

### 3.5 MRI acquisitions

The TDD‐MRI was implemented as three PGSE DW‐MRI acquisitions with different diffusion times, using spin echo EPI readout and sequence parameters listed in Table 1. Two clinical 1.5 T MRI systems were used to acquire TDD‐MRI: a conventional 1.5 T MRI system (Ingenia 1.5 T, R5.7.1, Philips HealthCare, Best, the Netherlands) with maximum gradient strength *G*_max_ = 45 mT/m and a 16‐channel head coil, and a 1.5 T MRI‐Linac system (Elekta Unity 1.5 T, R5.7.1, Elekta Solutions AB, Stockholm, Sweden) with *G*_max_ = 15 mT/m and system‐specific 4‐channel anterior and posterior coils. Asparagus was imaged with both systems while patients were imaged only with the conventional 1.5 T MRI system. The acquisition time for one diffusion time for the patient scans was 06:12. The TDD‐MRI acquisition used the MRI pulse sequence editing tools (Sequence Development Editor) available on Philips systems under a research software option. From patients, conventional MRI including 3D T1‐weighted gadolinium‐enhanced (T1w Gd) and 3D T2‐weighted FLAIR gadolinium‐enhanced (T2w FLAIR Gd) images were acquired with parameters defined in Table S1.

**TABLE 1:** TDD‐MRI acquisition parameters.

	1.5 T MRI‐Linac, *G*_max_ = 15 mT/m	Conventional 1.5 T MRI, *G*_max_ = 45 mT/m	
**Parameters**	Biological phantom (asparagus)	Biological phantom (asparagus)	Patients
*b* (no. averages) (s/mm^2^)	0 (2), 250 (4), 500 (8), 750 (8), 1000 (8)	0 (2), 1250 (6), 2500 (6)	0 (2), 1250 (6 or 8), 2500 (6 or 8)
*t_d_* (ms)	36, 44, 61	26, 44, 80	26, 44, 80^a^
*δ*/*∆* (ms)	42, 54, 42/50, 62, 75	27, 54, 27/35, 62, 89	27, 54, 27^a^/35, 62, 89^a^
*N*_dir_	6	6	6
TE/TR (ms)	135/10 000	135/10 000	135/3 756
In‐plane voxel size (mm)	3 x 3	3 x 3	3 x 3
Slice thickness (gap) (mm)	5 (1)	5 (1)	5 (1)
FOV (mm)	230 x 230 x 131	230 x 230 x 131	230 x 230 x 131

*Note*: The listed *t_d_*, δ and ∆ values correspond to the optimized waveforms (WF_1_, WF_2,_ and WF_3_) determined based on simulations.

Abbreviations: ∆, diffusion gradient separation; *b*, *b*‐value (amount of diffusion weighting); FOV, field of view; N_dir_, number of diffusion gradient directions; *t_d_*, effective diffusion time; TE, echo time; G_max_, maximum gradient strength; TR, repetition time; δ = diffusion gradient duration.

^a^ Not used on Patients A and B.

### 3.6 Data analysis

The TDD‐MRI data were corrected for subject motion and eddy current‐induced geometrical distortions using an open source framework for analysis of diffusion MRI data^61^ and Elastix (Elastix 5.1.0, Image Sciences Institute, University Medical Center Utrecht, Utrecht, The Netherlands).^62^ The framework uses extrapolated reference images for the distortion correction.^63^ Subsequently, susceptibility correction was performed using the TOPUP tool from FMRIB Software Library (FSL 6.0.5.2, Analysis Group, FMRIB, Oxford, UK).^64^, ^65^ The DW‐MRI signals for the TDD contrast calculations were the geometric means over the six diffusion directions, corrected for potential background gradients following a previously suggested procedure.^66^ To account for potential differences in signal gain between the different acquisitions and different T2 relaxation times, each acquisition was normalized with the *b* = 0 s/mm^2^ image. Lastly, the data were smoothed using a 3D median filter using half of the acquisition voxel size as the kernel size. The TDD contrast maps were calculated using Equation (2) for each *b*‐value. The ADC maps were calculated separately for each *t_d_* using all *b*‐values by fitting a log‐linear model to the DW‐MRI signal voxel‐wise. The code for processing and analyzing the data was written in Matlab (R2022a, MathWorks Inc., Natick, MA, USA).

Asparagus ROIs were drawn on the *b* = 0 s/mm^2^ image. The mean TDD signal within the asparagus ROIs was calculated after excluding data points more than 1.5 interquartile ranges above the 75th percentile or similarly below the 25th percentile.

For patients referred to RT, the gross tumor volume (GTV) was delineated on the T1w Gd images by an oncologist and a radiologist in collaboration. For the patients referred to surgery, the initial delineations of the contrast‐enhancing tumor volumes (delineated by M.J.) were reviewed and corrected by a radiologist (F.S.G.H.) with 8 years of expertise in neuroradiology. For simplicity, we denote all tumor delineations with the abbreviation GTV henceforth. The GTVs were propagated to the TDD contrast maps and other structural MR images using rigid registrations obtained between the different images in Elastix. The structural MR images and ADC maps were reviewed by the radiologist.

### 3.7 Noise estimation

To estimate the noise level in the DW‐MRI scans, six patients and one healthy volunteer were scanned with repeated *b* = 0 s/mm^2^ scans (*N*_repetitions_ = 10 for patients, *N*_repetitions_ = 20 for volunteer) with acquisition parameters corresponding to the patient TDD‐MRI protocol (Table 1). After eddy current, motion and susceptibility corrections, the remaining noise level was estimated as the voxel‐wise standard deviation over the repeated scans, which was averaged over the white matter (all subjects) and GTV (patients) ROIs. The white matter ROI was segmented with FMRIB's Automated Segmentation Tool (FSL 6.0.5.2, Analysis Group, FMRIB, Oxford, UK).^64^, ^67^ These noise level estimates and the measured mean signal levels in the TDD‐MRI images were used to simulate noisy signals by Monte Carlo sampling. The simulated signals were then processed like real TDD‐MRI data to estimate the amount of noise in TDD contrast maps. While the standard deviation across repeated scans is not a direct estimate of the thermal noise, we still refer to this as the estimated thermal noise, because thermal noise from the subject and the MRI scanner electronics is likely to be the dominating cause of variation in the *b* = 0 s/mm^2^ acquisitions after the motion and distortion corrections.^68^ The detailed noise estimation procedure is documented in Section S5.

## 4 RESULTS

### 4.1 Simulations

**FIGURE 3:**
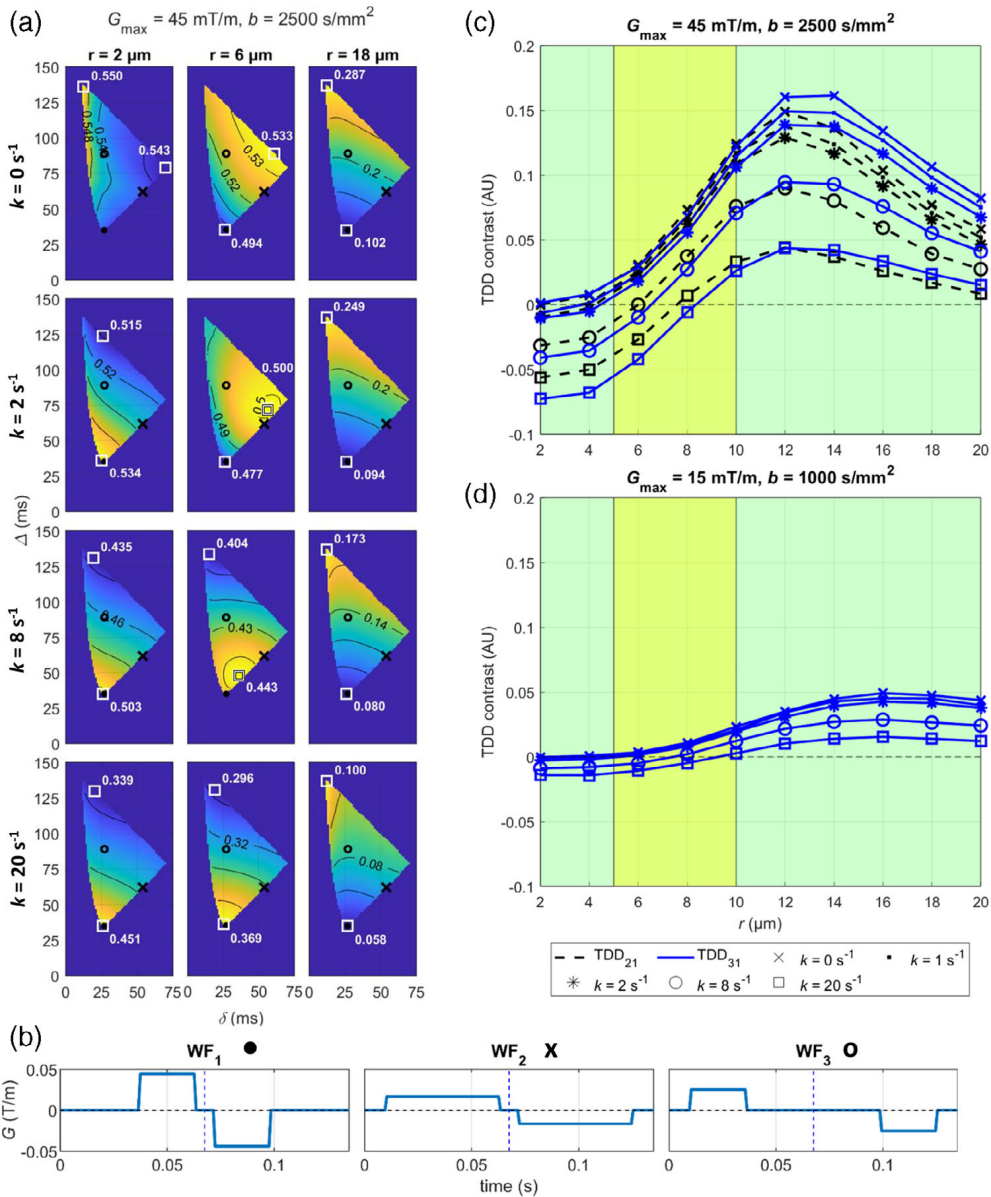
DW‐MRI signal and TDD contrast behavior in simulations. (a) Maps showing the DW‐MRI signal for gradient waveforms with multiple *δ*/*∆* combinations in 12 different tissue microstructures (tiles). The color scale is different in each tile, allowing comparison of the direction of the signal gradient in different microstructures. The absolute signal values are shown with the isocontours. The locations of the maximum and minimum signals are marked with white squares. The three optimized gradient waveforms are marked with a black dot (WF_1_), a black cross (WF_2_), and a black circle (WF_3_). To maintain a sufficient signal‐to‐noise ratio in the actual measurements, the maximum echo time was restricted to 135 ms for the optimized waveforms (simulations were performed with echo times up to 155 ms). (b) Schematic representation of the optimized gradient waveforms for *G*_max_ = 45 mT/m, *b* = 2500 s/mm^2^. Waveforms for *G*_max_ = 15 mT/m, *b* = 1000 s/mm^2^ are shown in Figure S1. (c and d) The theoretical TDD contrast (AU) with the optimized waveforms. The green background color indicates possible cell sizes for asparagus cells,^46^ while the yellow background color indicates typical sizes for tumor cells.^15^, ^40^ AU, arbitrary units; DW‐MRI, diffusion‐weighted magnetic resonance imaging; TDD, time‐dependent diffusion.

The simulations revealed two principal methods to maximize the TDD contrast. With no exchange, the TDD contrast was maximized with varying gradient duration (*δ*) in small restriction sizes (*r* ∈]2,10] µm), whereas in the case of larger restriction sizes varying gradient separation (*∆*) provided the contrast (Figure 3a for *G*_max_ = 45 mT/m, *b* = 2500 s/mm^2^). In the smallest of cell sizes (*r* = 2 µm), the contrast was also maximized with *δ*, but with an opposite signal gradient compared to restriction sizes *r* ∈]2,10] µm. Contrast related to diffusional exchange also depended on *∆*. Similar signal behaviors were seen with *G*_max_ = 15 mT/m (Figure S1), although the signal differences achievable with the lower gradient strength were smaller and the number of feasible gradient waveforms was lower. Three optimized waveforms WF_1_, WF_2_, and WF_3_ (Figure 3b) produced maximal TDD contrast either due to a change in *δ* (TDD_21_ between WF_2_ and WF_1_) or due to a change in *∆* (TDD_31_ between WF_3_ and WF_1_), were chosen for further investigation. The exact values for *δ*, *∆*, and *t_d_* for these waveforms are listed in Table 1. Figure S2 in supplementary material shows the simulated DW‐MRI signal decays with the optimized waveforms in a few example geometries.

The simulated TDD contrasts increased up to cell size *r* ≈ 12 µm for *G*_max_ = 45 mT/m and up to *r* ≈ 16 µm for *G*_max_ = 15 mT/m, and decayed for larger cells (Figure 3c,d). The maximum TDD contrasts due to restricted diffusion were 16.14% for *G*_max_ = 45 mT/m, *r* = 12 µm, and 4.91% for *G*_max_ = 15 mT/m, *r* = 16 µm, and were achieved with TDD_31_ for both gradient strengths. For *G*_max_ = 15 mT/m, only the TDD_31_ is shown, as the TDD_21_ contrast behaved similarly but had a lower magnitude. Slow diffusional exchange (*k* = 1, 2 s^−1^) dominated the contrast only in cell sizes *r* < 5 µm, whereas a faster exchange (*k* = 8, 20 s^−1^) could dominate the measurement up to cell sizes *r* ≈ 6–8 µm with *G*_max_ = 45 mT/m and *r* ≈ 8–10 µm with *G*_max_ = 15 mT/m and also diluted the TDD contrasts in larger restriction sizes. In general, the simulated exchange rates *k* = 1, 2, 8, and 20 s^−1^ decreased the contrast on average 0.77%, 1.44%, 4.54%, and 7.99% with *G*_max_ = 45 mT/m and 0.22%, 0.42%, 1.38%, and 2.34% with *G*_max_ = 15 mT/m. Within a typical cancer cells size range (*r* = 5–10 µm),^15^, ^40^ the TDD_21_ contrast emphasized the restriction effects in the presence of exchange, whereas in larger cells the TDD_31_ contrast was less diluted by the exchange. The maximum absolute TDD contrast due to diffusional exchange (i.e., the most negative contrast) was 7.28% with *G*_max_ = 45 mT/m but only 1.42% with *G*_max_ = 15 mT/m and was also achieved with TDD_31_ in the smallest cell size (*r* = 2 µm). A varying cell density did not change the contrast behaviors described above, but a higher cell density increased the TDD contrast (Figure S3).

### 4.2 TDD contrast in asparagus‐water phantom

**FIGURE 4:**
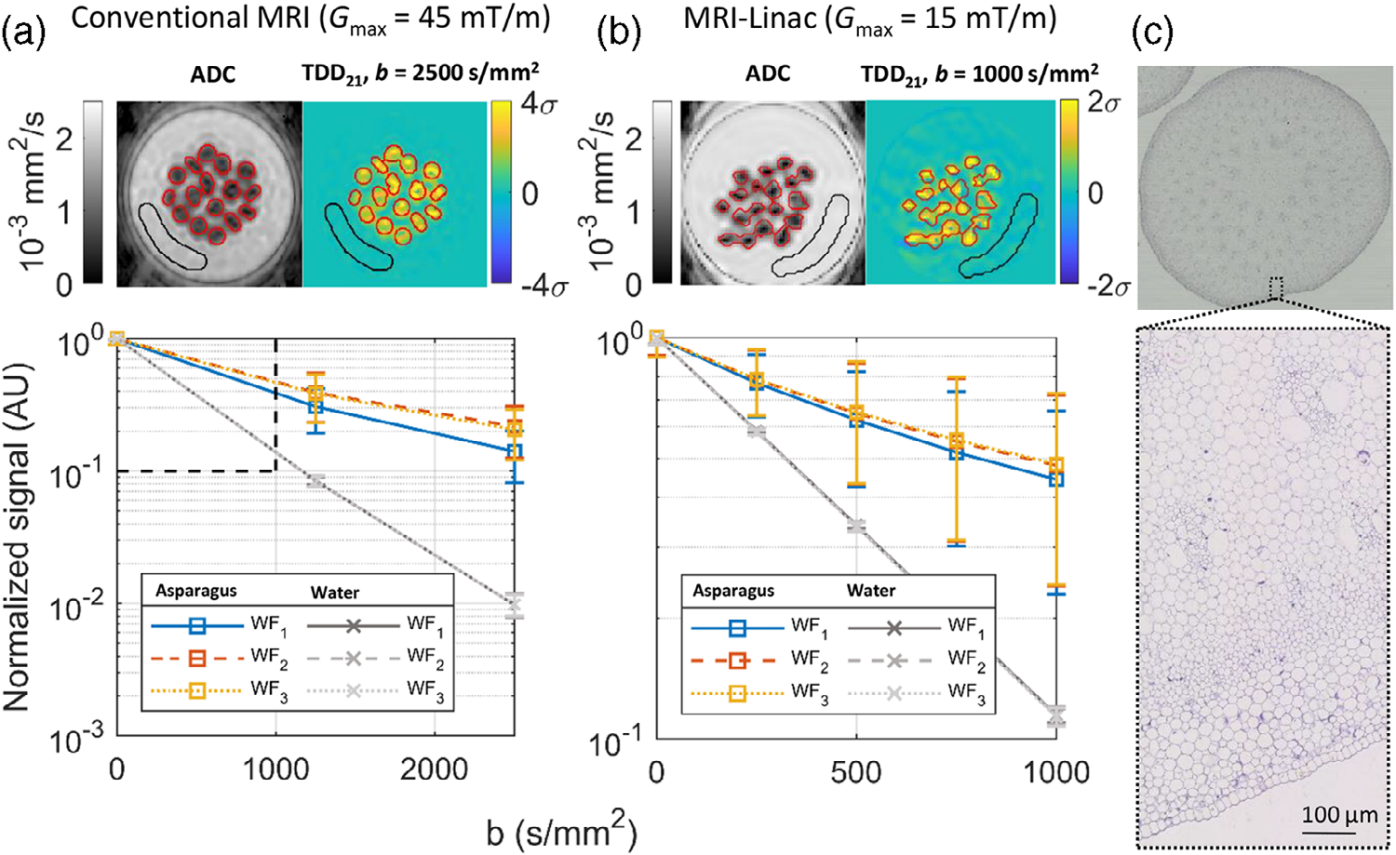
Diffusion time dependence in asparagus. (a) Results with the conventional MRI showing the ADC map (left), example TDD_21_ contrast map (right), and the mean DW‐MRI signal with the three gradient waveforms in asparagus and water ROIs (bottom). The asparagus ROI (red) and the water ROI (black) are shown in the ADC map and the TDD_21_ contrast map. The dashed box in the bottom panel shows the plotting area for the MRI‐Linac data. The error bars in the bottom panels correspond to ± 1σ. (b) Same as (a) but for the MRI‐Linac. (c) Optical microscopy of asparagus stems with 1 × (top) and 10 × (bottom) magnification. ADC, apparent diffusion coefficient; DW‐MRI, diffusion‐weighted magnetic resonance imaging; ROI, region of interest; TDD, time‐dependent diffusion.

With both MRI systems, the TDD contrast maps showed a strong positive signal in asparagus ROIs, indicating dominating restricted diffusion compared to the water ROIs, where no time‐dependent effects were measured (Figure 4, Figure S4). TDD_21_ and TDD_31_ contrasts were similar for both MRI systems reflecting the simulation results for larger restrictions without exchange in Figure 3a. After outlier exclusion (Table S2), the largest mean contrast within the asparagus ROI was 7.82% ± 3.77% with the conventional MRI (achieved with TDD_21_, *b* = 1250 s/mm^2^, *G*_max_ = 45 mT/m) and 3.05% ± 2.19% with the MRI‐Linac (TDD_31_, *b* = 1000 s/mm^2^, *G*_max _= 15 mT/m). The measured mean cell radius based on histology was 22.73 µm in the central part of the asparagus and 9.40 µm in the periphery, and the TDD contrast maps showed some indication of a decrease in the contrast from periphery to the center of the asparagus stems.

### 4.3 TDD contrast in patients

Ten patients were included with characteristics reported in Table 2. Three patients had been operated (Patients A and C: gross tumor resection; Patient B: stereotactic biopsy) before the inclusion and acquisition of the study MRI scans. Otherwise, the patients were MRI scanned for the study before receiving treatment.

**TABLE 2:** Summary of patient characteristics.

Age (in years, mean ± std, [range])		57.5 ± 12.4 [36‐72]
Sex	Male	6
	Female	4
Diagnosis	Glioblastoma, IDH‐wildtype (WHO 4)	7
	Lower grade glioma (WHO 3): MGMT methylated glioma, IDH mutated	1
	Metastasis (ovarian cancer)	2
Referral at the time of inclusion	Gross tumor resection	5
	Stereotactic needle biopsy	1
	Radiotherapy	4

**FIGURE 5:**
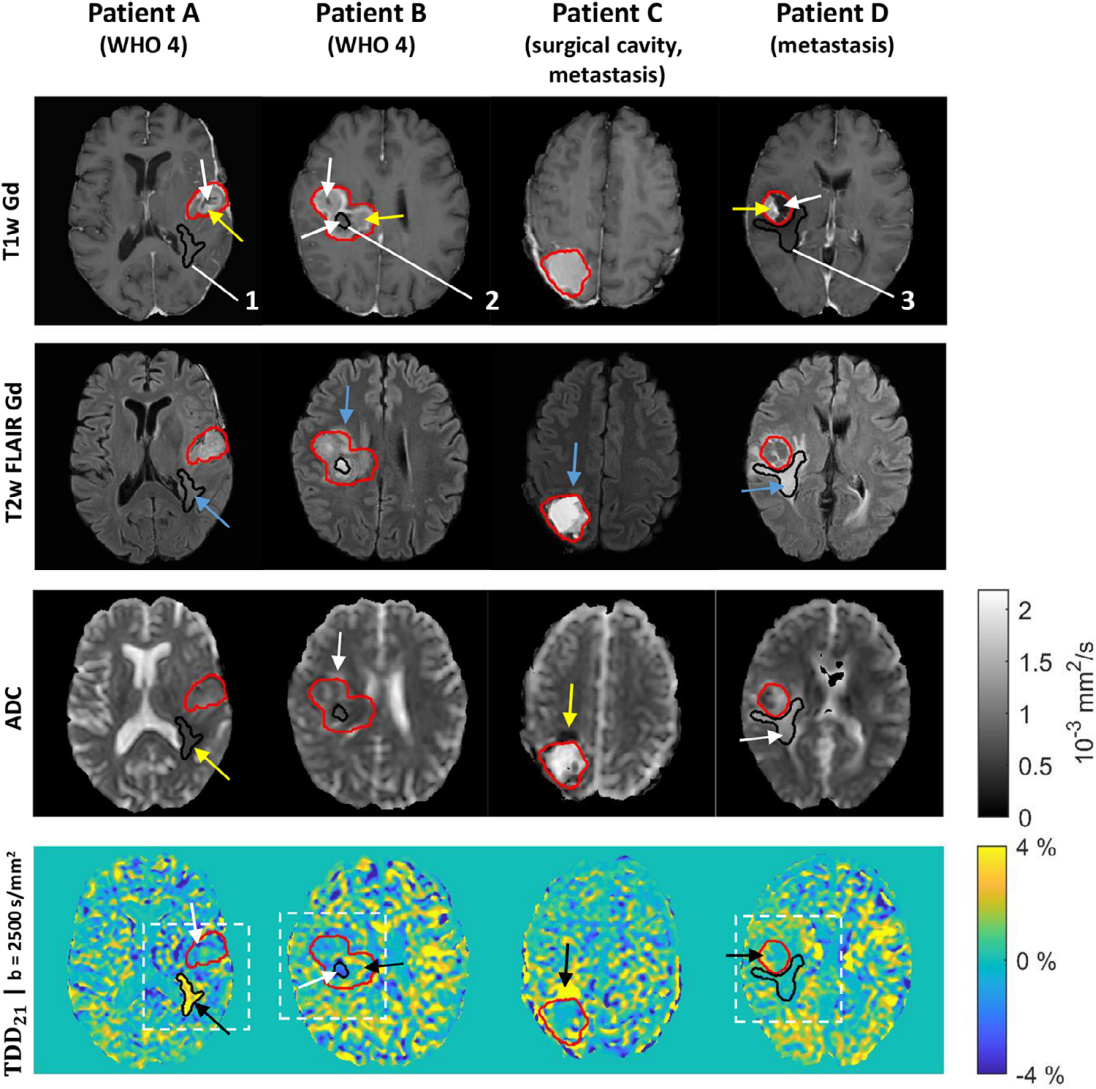
The first four patient cases. The GTV (red contour) and three example ROIs (black contours with labels 1–3) are shown on T1w Gd and T2w FLAIR Gd images, ADC map, and TDD_21_ *b* = 2500 s/mm^2^ contrast map. The arrows in the two topmost rows indicate different regions in the tumors: viable tumor mass (yellow arrows), necrotic core (white arrows), and peritumoral T2w FLAIR Gd hyperintensity regions (blue arrows). The T2w FLAIR Gd hyperintensity had either low ADC values (yellow arrows) or high ADC values (white arrows). The TDD contrast maps showed areas of positive contrast (black arrows), indicating dominating restricted diffusion, and areas of negative contrast (white arrows), indicating dominating diffusional exchange. White dashed boxes in TDD contrast maps indicate the zoom‐in regions for Figure 6. ADC, apparent diffusion coefficient; GTV, gross tumor volume; T1w Gd, T1‐weighted gadolinium contrast; T2w FLAIR Gd, T2‐weighted FLAIR gadolinium contrast; TDD, time‐dependent diffusion; ROI, region of interest; WHO 4, World Health Organisation grade 4 glioma.

In the structural MRI (Figure 5, Figure S5), all tumors showed contrast‐enhancing viable tumor mass in the T1w Gd images (yellow arrows), which surrounded a non‐enhancing necrotic core (white arrows), except for Patient I which only showed a small contrast enhancing region without a necrotic core (Figure S5). T2w FLAIR Gd hyperintense peritumoral regions were seen in all patients (blue arrows). For Patients A and C, these regions were hypointense in the ADC map (yellow arrows) and were assumed to be a region of infiltrative edema (Patient A) and a region of ischemia (Patient C) based on the tumor type. For all other patients, the T2w FLAIR Gd hyperintense regions were also hyperintense in the ADC map (white arrows), indicating vasogenic edema. The surgical cavity (Patient C) was mainly filled with liquid with some remaining blood products. Hemorrhage was also seen in Patients A and B due to previous invasive procedures. The ADC maps indicated low to intermediate diffusivity in the contrast‐enhancing tumor regions as well as in areas of hemorrhage and high diffusivities in necrotic regions and the surgical cavity.

**FIGURE 6:**
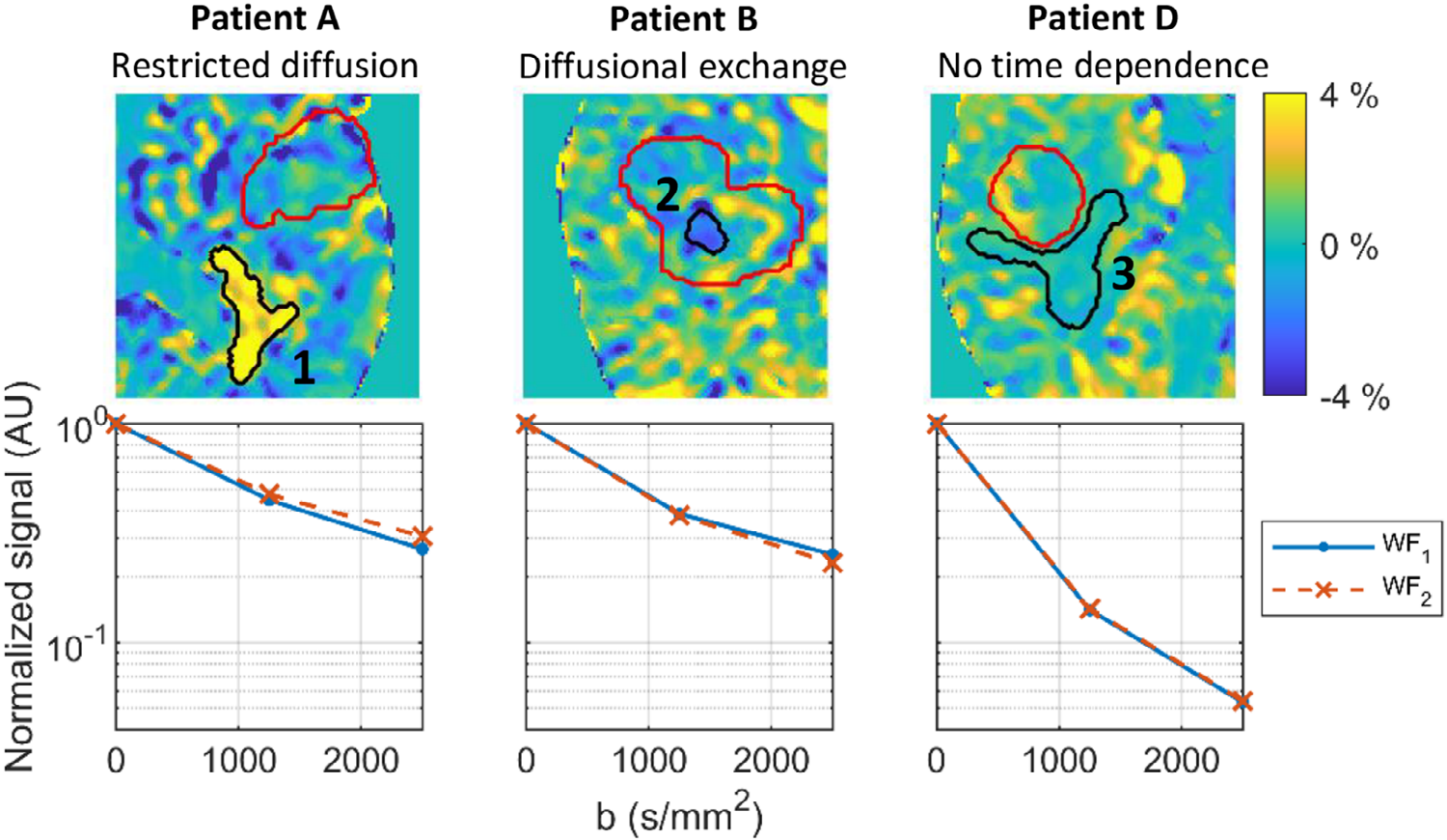
Examples of diffusion time dependence in brain lesions. The top row shows the magnifications of three regions defined in Figure 5. The black ROIs show examples of dominating restricted diffusion (ROI 1), dominating diffusional exchange (ROI 2), and no time dependence (ROI 3). The red contours indicate the location of the GTVs. The bottom row shows the ROI‐averaged DW‐MRI signals within ROIs 1–3 measured with two diffusion times (WF_1_: *t_d_* = 26 ms, WF_2_: *t_d_* = 44 ms) demonstrating the diffusion time dependence behind the different contrasts. DW‐MRI, diffusion‐weighted magnetic resonance imaging; GTV, gross tumor volume; ROI, region of interest.

The thermal noise level of TDD contrast maps was estimated to be 0.43% ± 0.15% in GTV, and 0.71% ± 0.10% in white matter (Figure S6, Tables S3‐S6). Persisting features could be seen in the TDD contrast maps across the 10 patients and for both *b*‐values, although the TDD effects were amplified at the higher *b*‐value. The largest lesions and the peritumoral regions were distinguishable from the surrounding tissue as areas of more consistent signal and showed sub‐regions of positive (black arrows) and negative (white arrows) TDD contrast. Three examples of these sub‐regions (ROIs 1–3) are shown in Figure 5 (black contours). The relation between the TDD contrast and other MR image contrasts in these three ROIs is listed in Table 3. The DW‐MRI signal behavior for WF_1_ and WF_2_ is shown in Figure 6 within the three example ROIs. The signal decays demonstrate examples of dominating restricted diffusion (ROI 1), dominating diffusional exchange (ROI 2), and no time dependence (ROI 3). TDD_21_ and TDD_31_ contrasts were visually compared in Patients C and D (Figure S7) and deemed to be similar, as in the case of asparagus (Figure 4).

**TABLE 3:** Contrasts in TDD contrast maps, ADC maps, and structural MR images within three example ROIs (1–3) shown in Figure 5.

	TDD	ADC	T2w FLAIR Gd	T1w Gd
**ROI 1**	+	hypo	hyper (moderate)	iso
**ROI 2**	–	hyper (moderate)	hyper	hypo
**ROI 3**	0	hyper	hyper	hypo

*Note*: For the ADC and structural MR images, signal intensity in white matter was the reference.

Abbreviation: —, negative TDD contrast; +, positive TDD contrast; 0, no TDD contrast; ADC, apparent diffusion coefficient; hyper, hyperintense; hypo, hypointense; iso, isointense; ROI, region of interest; TDD, time‐dependent diffusion.

## 5 DISCUSSION

In this feasibility study, we implemented TDD‐MRI on MRI systems with limited gradient strengths, which are common in RT departments. We used a simple analysis framework to quantify the available contrast in the source DW‐images to evaluate whether restricted diffusion or diffusional exchange effects are dominating when measured on these systems. The implemented TDD contrast maps are comparable across subjects similarly to more sophisticated biophysical models but are free from model parameter assumptions, and could provide supplementary information to ADC maps already established in tumor characterization. Similar approaches have been investigated before but the investigations have focused on axon and gray matter soma diameter mapping and used stronger gradient strengths (*G*_max_ ≥ 80 mT/m),^39^ or have been done on animals.^29^

Simulations over a range of restriction sizes and exchange rates showed that the maximum contrast was achieved by maximizing either gradient duration (*δ*) for small restrictions, or separation (*∆*) for larger restrictions and diffusional exchange. The *δ*‐dependent contrast (TDD_21_) in small restrictions has been demonstrated before in studies focusing on axon diameter mapping,^39^, ^42^, ^69^ and can be explained by the COM effect. The *∆*‐dependent contrast (TDD_31_) in larger restrictions indicated that these restriction sizes were sufficiently large to be insensitive to the COM effect. The smallest simulated restriction size (*r* = 2 µm) demonstrated a third kind of contrast (Figure 3a, top left corner). This weak effect was verified to originate from the connected porous geometry of the extracellular space (data not shown).

The simulations demonstrate, how the TDD contrast is able to differentiate only dominating diffusional exchange in smaller cells from dominating restricted diffusion in larger cells. The only “pure” TDD effect a simple PGSE experiment can measure is diffusional exchange when restriction sizes are too small for the experimental parameters to detect restriction‐related signal changes. Using a previously presented formula for this *resolution limit*,^42^ we obtain *r* = 2.9 µm and *r* = 4.5 µm for our measurements with *G*_max_ = 45 mT/m and *G*_max_ = 15 mT/m, respectively, assuming a noise level of 2%. The formula is made for cylinders, which means that it estimates slightly better resolution compared to the spherical geometries used in our study. The result is in line with previous studies, which have estimated the resolution limit to be *r* = 2–4 µm for *G*_max_ = 60–80 mT/m.^42^, ^69^ The estimated resolution limit for our measurements is high compared to normal‐appearing brain tissue, where typical cell sizes vary from below *r* = 1 µm (axons).^41^, ^70^ However, the estimates for both gradient strengths were still below the typical cell size range for cancer cells (*r* = 5–10 µm),^15^, ^40^ indicating that the TDD contrast would be sensitive around a clinically relevant cell size range for cancer applications.

Above the resolution limit and within the cell size range typical for cancer cells the simulated TDD contrast due to restricted diffusion varied between 2% and 12% for *G*_max_ = 45 mT/m, but barely exceeded 2% with *G*_max_ = 15 mT/m (Figure 3c,d). This means that the TDD contrast with gradient strengths corresponding to the MRI‐Linac systems (*G*_max_ = 15 mT/m) might be in the same order as typical noise levels, whereas gradient strengths corresponding to the conventional MRI systems (*G*_max_ = 45 mT/m) could produce detectable contrast within a relevant cell size range for cancer applications. However, exchange rates from 8 to 20 s^−1^ efficiently diluted the contrast from restricted diffusion and even dominated the contrast (Figure 3c,d). Although typical exchange rates in tissue are subject to debate, exchange rates ranging from *k* = 6–8 s^−1^ up to *k* = 20 s^−1^ have been reported in tumor cells and in gray matter.^71^, ^72^, ^73^ Our results support recent findings on how exchange should be taken into account in tumor microstructure modeling.^28^ The potential relevance of exchange is possibly emphasized when measuring TDD effects with limited gradient strengths, as the inherently long *t_d_*:s could more easily overlap with the intracellular lifetime of water. This is against the general assumption that relevant restriction sizes are detected with considerably shorter *t_d_* than what is required for detection of diffusional exchange.^15^

Although the TDD contrast is inherently affected by both restricted diffusion and diffusional exchange, it can provide information of the interplay of these competing effects. The TDD contrast can help to measure the dominating TDD effect to guide selection of more sophisticated models, and help to avoid attempts of model fitting in situations, where the two effects cancel each other preventing any meaningful fitting. Calculating the TDD contrast requires just two measurements with a constant echo time and *b*‐value but with different *t_d_*:s, which means that it can be calculated from data collected for the principal biophysical models.^17^, ^18^, ^19^

The TDD contrast was further investigated in a phantom using materials previously demonstrated to exhibit Gaussian (water) and non‐Gaussian (green asparagus) diffusion on clinical systems.^46^, ^47^, ^48^, ^49^ Furthermore, although the primary objective of the phantom measurements was not to mimic tumor microstructure, asparagus cell sizes reported in the literature (*r* = 2–25 µm)^46^ roughly cover the range of expected cell sizes in tumors (*r* = 5–10 µm),^15^, ^40^ although the average cell size in asparagus is expected to be larger.^46^

The measurements in asparagus showed a positive TDD contrast with both clinical scanners (largest mean TDD contrasts 7.82% ± 3.77% and 3.05% ± 2.19% with the conventional MRI and MRI‐Linac, respectively). The contrast variation can relate to the differences in the ADC across the large asparagus ROIs, which covered both the periphery with smaller mean cell size and the center with larger mean cell size. We find it promising that the TDD contrast decreased from peripheral to central asparagus possibly reflecting the change in mean cell size despite the large voxel size. The results were in line with the simulations when taking into account that the simulations gave the maximum contrasts for individual cell sizes, whereas, in the asparagus experiment, we measured the mean TDD contrast over the range of cell sizes present in the asparagus.

Measurements in patients were only carried out on the conventional MRI system with *G*_max_ = 45 mT/m, as the TDD contrast in asparagus on the MRI‐Linac was low (< 5%) despite the relatively large mean cell sizes measured in asparagus.

The TDD contrasts seen in patients were generally much lower than in simulations or asparagus measurements, and the TDD contrast maps seemed noisy across the brain. However, while previous studies report low TDD effects in normal‐appearing neuronal tissue with moderate gradient strengths,^39^, ^72^ the TDD contrast maps within and around the GTVs and across patients contained consistent regions that were unlikely to be explained by noise alone. This supports our hypothesis that the larger mean cell size in tumors can help the detection of TDD effects with moderate gradient strengths compared to axon imaging. Furthermore, the thermal noise level was estimated to be lower in GTV versus white matter, which can be due to a higher T2‐weighted signal in tumors. This potentially lowers the contrast detection threshold in tumors compared to white matter, where high gradient strengths have been a necessity to produce detectable time‐dependent signal changes.^42^, ^69^ The lower TDD contrasts measured in patients were in line with the assumption of a smaller average tumor cell size (*r* = 5–10 µm)^15^, ^40^ compared to the cell sizes measured in asparagus (*r* = 9.40–22.73 µm).

The consistent sub‐regions in the TDD contrast maps partly corresponded to the visible tumor structures in the structural MR images. The vasogenic edemas with hyperintense T2w FLAIR Gd contrast and high ADC exhibited no TDD contrast (e.g., Patient D in Figure 5 and Patient H in Figure S5), which matches the assumption of more freely moving water in these regions. The TDD contrast within the GTVs was generally most positive near the edges of the GTV and partly corresponded to the contrast‐enhancing tumor rims present in the T1w Gd images. This kind of TDD contrast behavior was particularly seen in Patients B (Figure 5) and Patient F (Figure S5), although present also in others (e.g., Patients D, E, H, and J). This supports the hypothesis that the viable tumor volume with possibly densely packed cells is assumed to be at the edges of the tumor volume.^74^ On the other hand, the central parts of the tumor, which were largely hypointense or mildly contrast enhancing in the T1w Gd images, exhibited mainly negative or near zero TDD contrast, as was seen for example, in Patient F (Figure S5). This is in line with the assumption that tumor cores have either moderate cellularity or are necrotic.^74^ Based on the simulations, it was clear that the TDD contrast is lowered by lower cell densities and higher diffusional exchange, both of which might be more prominent in the necrotic tumor core than in the viable edges.^74^, ^75^ Finally, the TDD contrast maps seemed to be able to distinguish purely vasogenic edema from potential infiltrative edema (Patient A) and from potential ischemia (Patient C).

The patient results demonstrate the spatial heterogeneity of brain lesions, which has been highlighted before particularly in gliomas.^24^ However, previous TDD‐MRI research in brain lesions has primarily focused on modeling restricted diffusion.^22^, ^24^, ^76^, ^77^ Emerging studies focusing on healthy gray matter have shown the importance of including exchange in the microstructure modeling,^71^, ^78^ and exchange effects are likely to be no less relevant in tumor tissue, as membrane permeability has been shown to be a potential differentiator between normal and pathological tissue,^79^, ^80^ and tumor cores often contain necrotic areas where cell membranes are disrupted.^75^ Our results suggest that diffusional exchange should be taken into account when modeling tissue microstructure in brain tumors, especially if tumors are expected to have necrotic regions.

The presented TDD contrast has two principal limitations. First, as discussed in relation to the simulation results, both restricted diffusion and diffusional exchange effects are mixed in the TDD contrast. A recent trend in TDD‐MRI is to design specific gradient waveforms that are sensitive to only one aspect of tissue microstructure at a time.^81^ Solutions for disentangling restricted diffusion from diffusional anisotropy have been particularly investigated.^82^ Solutions for diffusional exchange have also emerged, but continue to rely on the assumption that restriction and exchange effects occur within different timescales.^83^ Furthermore, complex gradient waveforms currently require special software solutions for implementation, which can hinder the clinical use of such acquisitions.

Second, the measured TDD contrast was low in the patient experiments. In principle, the TDD contrast could be increased with a larger change in *t_d_*. A feasible maximum *t_d_* is limited by the T2 relaxation, while the available gradient strength and desired *b*‐value determine the minimum *t_d_* in a PGSE experiment. The use of oscillating gradients with a limited number of oscillations could allow a shorter *t_d_* while maintaining, or even increasing, the *b*‐value.^15^ Although the current study focused on using conventional PGSE sequences, future studies should investigate the available TDD contrast with OGSE. A higher maximum gradient strength would also increase the TDD contrast.^39^ The increasing understanding of the potential of DW‐MRI for characterizing tissue microstructure in health and disease has already led to the development of high gradient strength systems, such as the Connectome system for brain imaging.^84^ The potential of biologically guided RT^1^, ^2^ could also justify a need for higher gradient performance systems in RT settings.

An alternative approach to increase the robustness of the current TDD contrast would be to reduce the noise. The current thermal noise investigation indicated that other noise sources should be investigated. Potential noise sources include spatial misalignment of the source DW‐MRI images and physiological noise, for example, due to blood flow. Although our analysis used an image registration and eddy current correction method, which has been shown to reduce systematic registration errors,^63^ and the TDD maps were checked for potential systematic effects on tissue borders, it was difficult to rule out the possibility of spatial misregistration completely. Future studies could consider exploring cardiac gating or interleaved acquisition of the different diffusion times to reduce the influence of physiological motion related to blood flow.

A further limitation of the current study is that we investigated the theoretical TDD contrast in a limited number of simple tissue geometries with constant cell sizes, cell densities, and exchange rates. In reality, the cell sizes in tissue are a distribution, which would affect the TDD contrast, as described before with a similar approach on high gradient strengths.^39^ Furthermore, the simulations were performed without added noise. Future studies should address this in order to study the noise floor in TDD‐MRI acquisition.

The sample size of the current study was limited and heterogeneous regarding the lesion type. Although the latter was intentional to investigate the TDD contrast in a range of tissue microstructures, larger sample sizes with histopathological ground truth are required to validate the TDD contrast. Furthermore, the reproducibility of the TDD contrast should be investigated before proceeding into clinical applications. In line with existing TDD studies in brain,^22^, ^30^, ^76^, ^85^ the current study was the necessary first step to investigate the feasibility of the TDD contrast in a smaller sample before larger‐scale studies. Our findings show some promise for the TDD contrast to characterize the dominating TDD effects in spatially heterogeneous brain lesions, motivating further studies. Based on our preliminary findings, information from TDD contrast maps could complement information from other MRI, for example, in treatment response monitoring, where repeated administration of gadolinium contrast during the treatment course might not be desirable, or in RT treatment planning to identify potential treatment resistant tumor sub‐regions. Previous research has suggested intensity‐based thresholding of ADC maps to estimate the viable tumor volume for RT treatment planning applications,^86^ and a similar approach could be considered with the TDD contrast.

## 6 CONCLUSIONS

In this study, we presented a TDD contrast framework and tested the feasibility of two clinical MRI systems. Based on simulations, the TDD contrast has the potential to distinguish dominating diffusional exchange in small cells from dominating restricted diffusion effects in larger cells and could provide information on which time‐dependent effects to include when choosing a biophysical model for more specific tumor characterization. On a conventional MRI system (*G*_max_ = 45 mT/m) the TDD contrast demonstrated consistent sub‐regions indicating both restricted diffusion and diffusional exchange within and across 10 brain lesions. On the MRI‐Linac (*G*_max_ = 15 mT/m) the TDD contrast showed the same trends as the results for the higher gradient strength in phantom measurements, but the available TDD contrast was possibly too low for reliable tissue microstructure characterization and was not further investigated in vivo. Future studies on conventional MRI systems should compare the TDD contrast to histology to validate the TDD contrast as a QIB for tumor characterization.

## CONFLICT OF INTEREST STATEMENT

H.L. is an inventor and has an interest in patents owned by RWI AB, Lund, Sweden, related to DW‐MRI methodologies not applied in this work. All other authors declare no conflicts of interest.

## Supporting information



Supplementary Information

## Data Availability

The datasets generated and/or analyzed during this study are available from the corresponding author upon reasonable request.
