# Supplementary material for: Assessing tumor microstructure with time‐dependent diffusion imaging: Considerations and feasibility on clinical MRI and MRI‐Linac
Source: Med Phys. 2024 Oct 10;52(1):346–61. doi: 10.1002/mp.17453 (PMC11700005; doi:10.1002/mp.17453)
Supplement: Supplementary file 1 — Supplementary Information [file MP-52-346-s001.pdf]

Supplementary material

Assessing tumor heterogeneity with time-dependent diffusion imaging: Considerations and feasibility on clinical MRI and MRI-Linac

Minea Jokivuolle[1,2*], Faisal Mahmood[1,2], Kristoffer Hougaard Madsen[3,4], Frederik Severin Gråe Harbo[5], Lars Johnsen[1], Henrik Lundell[3,6]

[1]Laboratory of Radiation Physics, Department of Oncology, Odense University Hospital, Odense, Denmark; [2]University of Southern Denmark, Department of Clinical Research, Odense, Denmark; [3]Danish Research Centre for Magnetic Resonance, Centre for Functional and Diagnostic Imaging and Research, Copenhagen University Hospital - Amager and Hvidovre, Copenhagen, Denmark; [4]Technical University of Denmark, Department of Applied Mathematics and Computer Science, Kongens Lyngby, Denmark; [5]Department of Radiology, Odense University Hospital, Odense, Denmark; [6]Technical University of Denmark, Department of Health Technology, Kongens Lyngby, Denmark;

*Corresponding author: Minea Jokivuolle

Address:
Laboratory of Radiation Physics, Department of Oncology, Odense University Hospital
Kløvervænget 19, 5000 Odense, Denmark
Email: minea.melissa.jokivuolle@rsyd.dk

1 Structural MRI scan parameters

Table S1. Acquisition parameters for the structural MRI scans. Abbreviations: T1w Gd = T1-weighted gadolinium contrast scan, T2w FLAIR Gd = T2-weighted Fluid-Attenuated Inversion Recovery gadolinium contrast scan, TE = echo time, TR = repetition time, TI = inversion time, SPIR = Spectral Presaturation with Inversion Recovery.

**Parameter**	**T1w Gd**	**T2w FLAIR Gd**
TE/TR/TI (ms)	shortest (≈ 4)/25	shortest (≈ 350)/4800/1660
Fat suppression	N/A	SPIR
Averages	2	3
In-plane voxel size (mm)	1 x 1	1 x 1
Slice thickness (mm)	2	3
FOV (mm)	230 x 170 x 150	230 x 170 x 150

2 Additional simulations

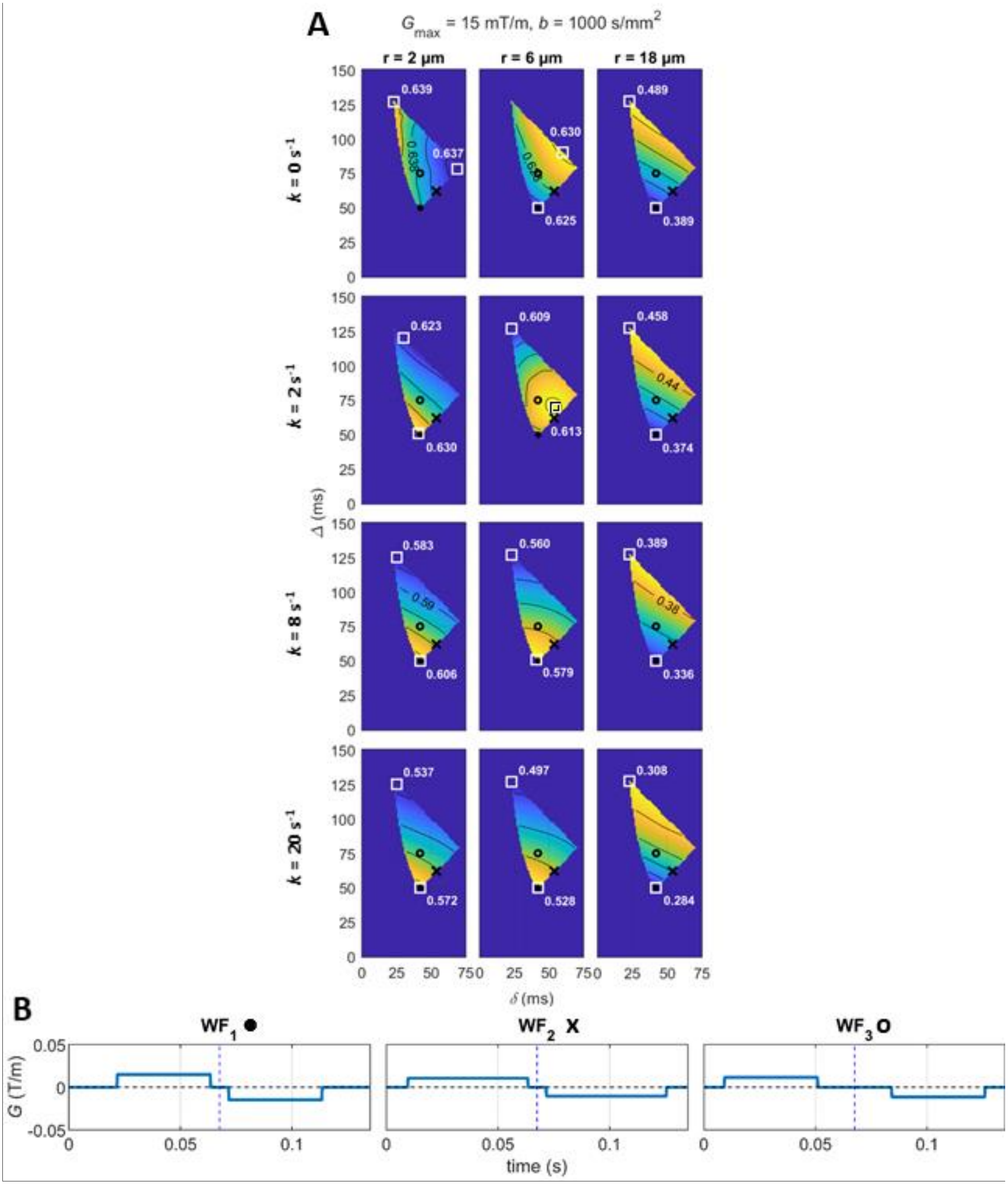

Figure S1. Simulations with the maximum MRI-Linac gradient strength. (A) Simulated diffusion signal for G_{max} = 15 mT/m with b = 1000 s/mm^2 in twelve different tissue microstructures (tiles). The maps show the diffusion weighted signal for gradient waveforms with multiple δ/Δ combinations. The color scale is different in each tile, allowing comparison of the direction of the signal gradient in different microstructures. The absolute signal values are shown with the isocontours. In addition, the location of the maximum and minimum signal is marked with white squares and the value shown in white. The three gradient waveforms chosen based on the simulation results for the actual measurements are marked with a black dot (WF_1), a black cross (WF_2) and a black circle (WF_3). (B) Schematic representation of the optimized gradient waveforms for G_{max} = 15 mT/m, b = 1000 s/mm^2.

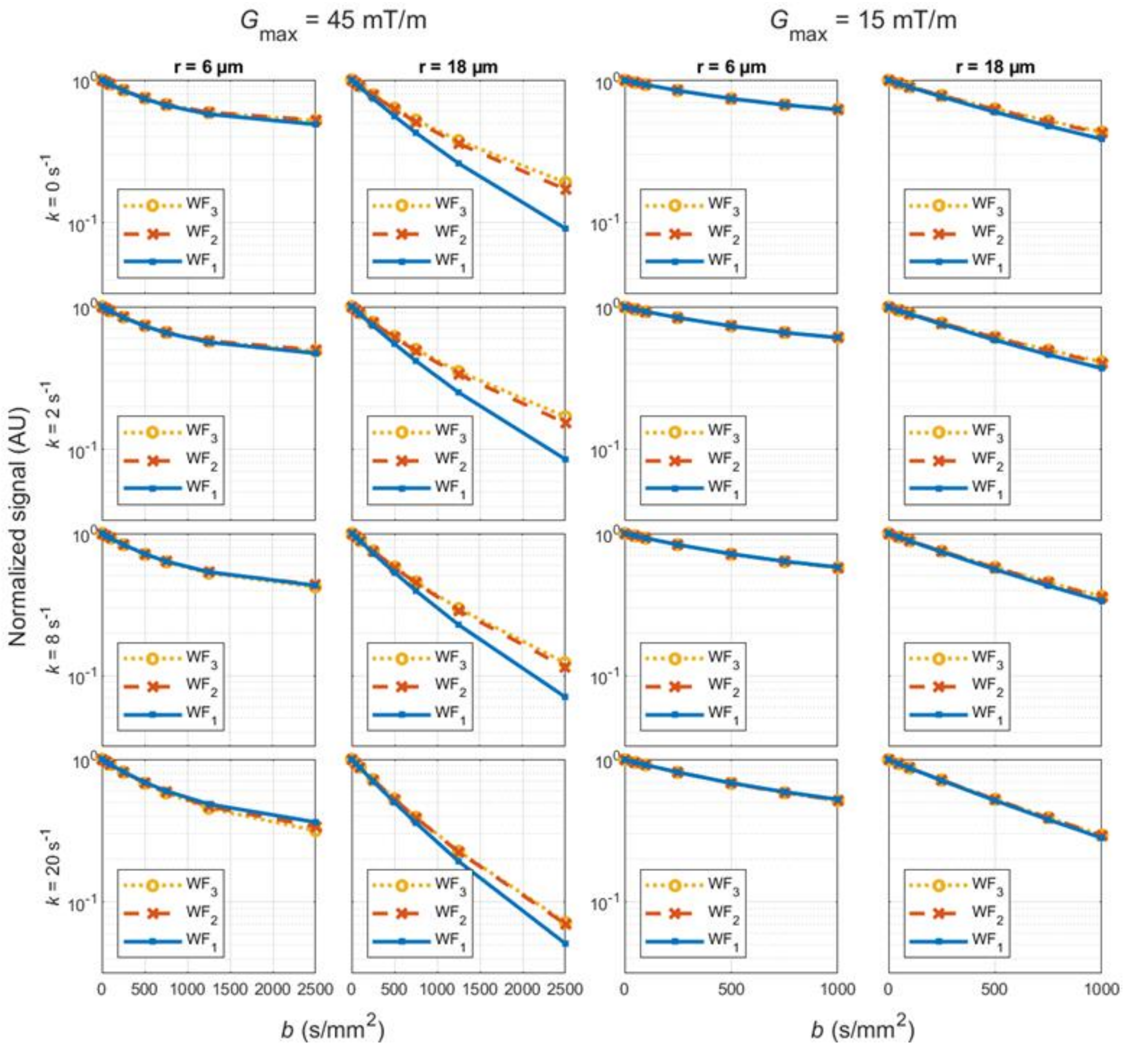

Figure S2. Simulated DW-MRI signal with the three optimized waveforms (WF_1, WF_2 and WF_3) for both gradient strengths (G_{max}) in eight example tissue geometries (tiles) with varying cell size (*r* = [6, 18] µm) and exchange rate (*k* = [0, 2, 8, 20] s^{-1}).

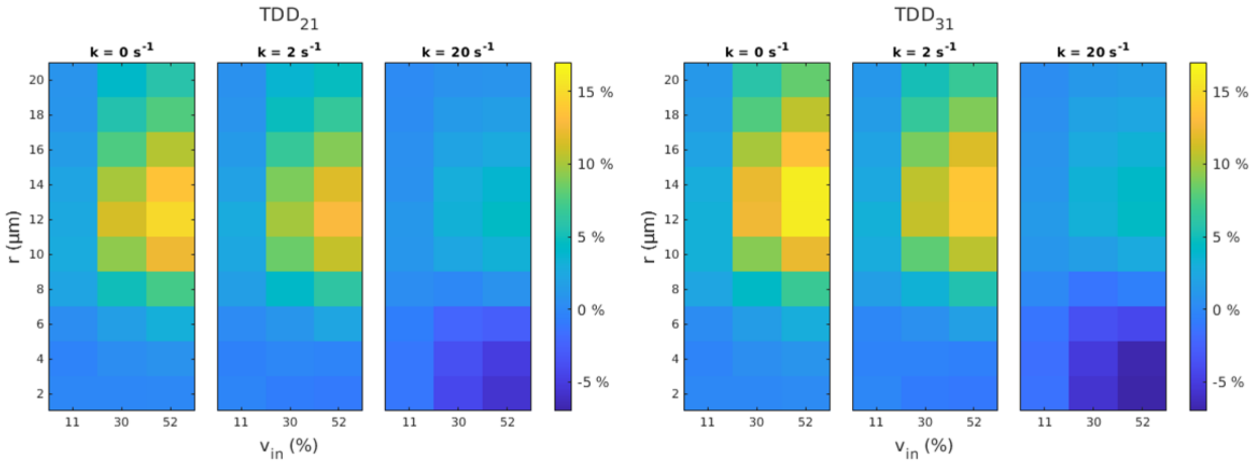

Figure S3. Simulations with varying cell density. The effect of cell density (v_{in}) to the TDD contrasts (left: TDD_{21} contrast, right: TDD_{31} contrast) with G_{max} = 45 mT/m, *b* = 2500 s/mm^2. Different cell sizes (*r*) and exchange rates (*k*) are also included.

3 TDD contrast distributions in phantom measurements

Outliers were removed from the TDD contrast distributions within the asparagus ROIs using the interquartile range criteria with the 'isoutlier' Matlab function (MATLAB R2022a, MathWorks Inc., Navick, MA, USA). The interquartile range for outlier detection is suited for skewed data, and was chosen after initial visualization of the complete data. In the method, outliers are defined as values more than 1.5 interquartile ranges above the upper quartile (75th percentile) or similarly below the lower quartile (25th percentile). Table S2 shows the number of excluded voxels per *b*-value for both scanners used in the measurements. Examples of the contrast distributions for the maximum *b*-values with both scanners are shown in Figure S4.

Table S2. Outliers in TDD contrast distributions within asparagus ROIs.

**Conventional MRI (G_{max} = 45 mT/m)**				
N_{voxels}	2084			
b (s/mm2)	1250		2500	
$N_{outliers}$	96 (4.61%)		76 (3.65%)	
**MRI-Linac (G_{max} = 15 mT/m)**				
N_{voxels}	482			
b (s/mm2)	250	500	750	1000
$N_{outliers}$	26 (5.39%)	29 (6.02%)	29 (6.02%)	24 (4.98%)

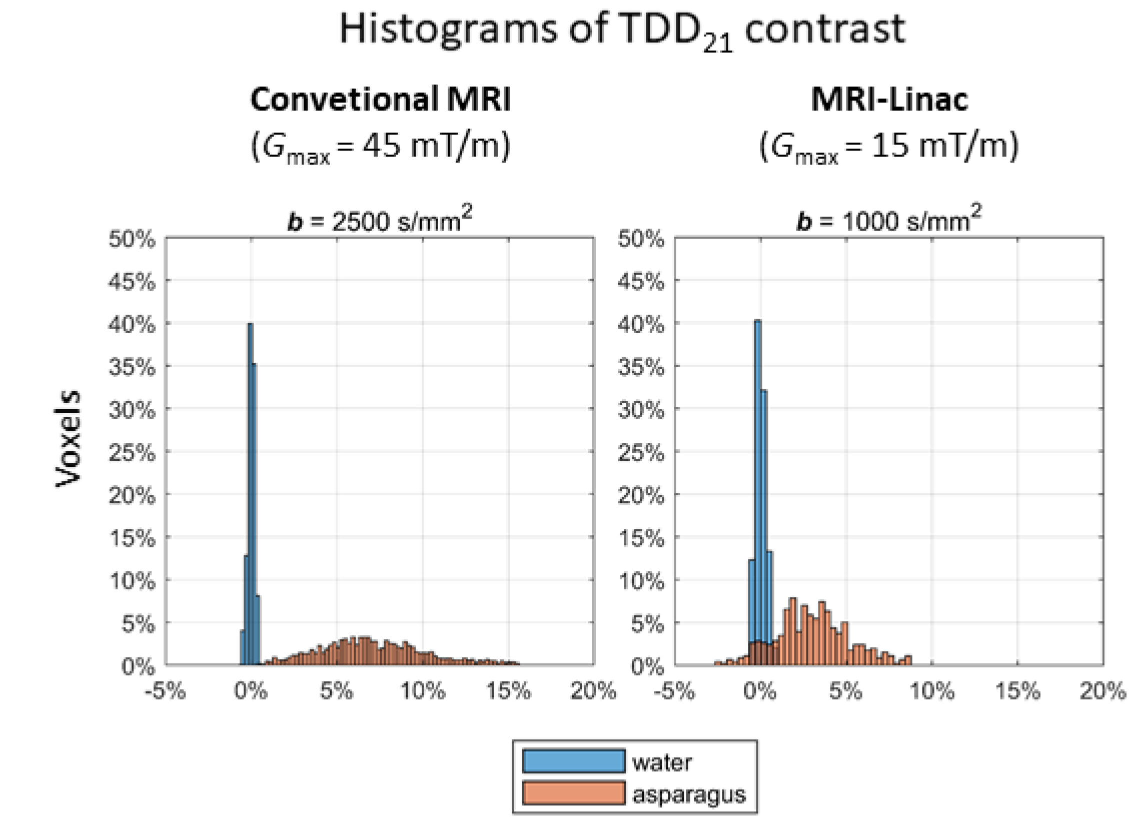

Figure S4. Histograms of TDD_{21} contrast. Distribution of TDD_{21} contrast values within the asparagus and water ROIs with the conventional MRI (G_{max} = 45 mT/m, *b* = 2500 s/mm^2, left) and with the MRI-Linac (G_{max} = 15 mT/m, *b* = 1000 s/mm^2, right) after removing outliers. The histograms were normalized with respect to the number of voxels per ROI.

4 Patient data

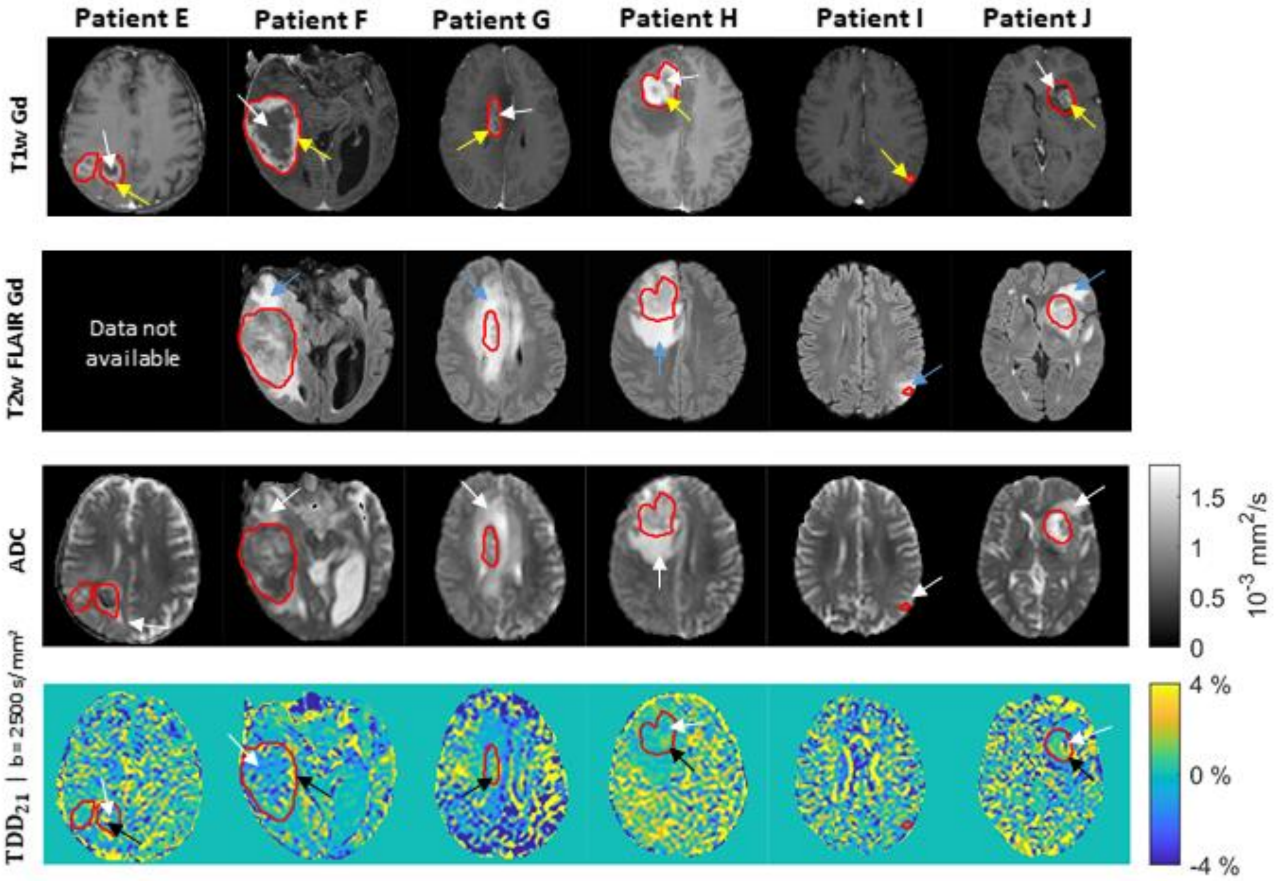

Figure S5. The remaining six patient cases. The GTV (red contours) are shown on T1-weighted gadolinium contrast (T1w Gd) and T2-weighted FLAIR gadolinium contrast (T2w FLAIR Gd) images, ADC map, and on TDD_{21} b = 2500 s/mm^2 contrast map. The arrows in the two topmost rows indicate different regions in the tumors: viable tumor mass (yellow arrows), necrotic core (white arrows), and peritumoral T2w FLAIR Gd hyperintensity regions (blue arrows). The T2w FLAIR Gd hyperintensity regions had high ADC values (white arrows). The TDD contrast maps showed areas of positive contrast (black arrows), indicating dominating restricted diffusion, and areas of negative contrast (white arrows), indicating dominating diffusional exchange.

5 Noise estimation

The amount of noise in the TDD contrast maps was estimated with the procedure described below. We refer to this estimated noise as thermal noise because thermal noise from the subject and the MRI scanner electronics is likely to be the principal cause of variation in the b = 0 s/mm^2 acquisitions after the motion and distortion corrections. [1]

Estimating thermal noise in the DW-MRI acquisition

1. A b = 0 s/mm^2 acquisition without averages was acquired repeatedly from six patients ($N_{\text{repetitions}}$ = 10) and a healthy volunteer ($N_{\text{repetitions}}$ = 20). Acquisition parameters were otherwise as in Table 1.
2. The repeated b = 0 s/mm^2 volumes were corrected for subject motion and eddy current as well as susceptibility induced geometric distortions as described in the manuscript.

3. A map of the thermal noise per subject was obtained as the voxel-wise standard deviation over the $N_{repetitions}$ (Figure S6). [2]
4. The white matter (WM) was segmented per subject from a T1-weighted image using FMRIB's Automated Segmentation Tool (FSL 6.0.5.2, Analysis Group, FMRIB, Oxford, UK). [3,4]
5. Gross tumor volume (GTV) for each patient was obtained as described in the manuscript Methods-section (delineated on the T1-weighted image).
6. One b = 0 s/mm^2 image was registered to the T1-weighted image using FMRIB's Linear Image Registration Tool (FSL 6.0.5.2, Analysis Group, FMRIB, Oxford, UK), [3,5,6] and the inverse transform was used to register the WM segmentation, i.e. the WM region of interest (ROI), and the GTV to the obtained noise map.
7. An estimate for the thermal noise level in the DW-MRI acquisitions was then obtained by averaging the noise map 1) within the WM ROI and 2) within the GTV (latter used only for patients).

The estimated absolute noise levels over the six patients were 84.36 in the WM ROIs and 98.57 in the GTV ROIs, which were 2.98% and 1.76% respectively of the measured b = 0 s/mm^2 signals. For the volunteer, the estimated absolute noise level was 77.59 in the WM ROI, which was 3.55% of the measured b = 0 s/mm^2 signal.

Estimating thermal noise in the TDD contrast maps

- Simulated DW-MRI signals were created in Matlab (R2022a, MathWorks Inc, Navick, MA, USA) with
    1. mean signal values corresponding to the actual measured mean signals within WM (Table S3) and the GTV (Table S4) for each patient,
    2. number of repetitions (diffusion directions and excitations) corresponding to the patient acquisition (acquisition parameters in Table 1, number of averages used in the simulation was six for the non-zero b-values),
    3. 100.000 “voxels” in each DW-MRI signal, and
    4. standard deviation corresponding to the noise level in the volunteer acquisition.
- The simulated signals were processed in the same way as actual DW-MRI measurements when creating TDD contrast maps, and the noise level in TDD contrast maps was estimated as the standard deviation over the simulated TDD contrast ‘voxels’ after visually ensuring that the distribution of the simulated TDD contrast voxels was roughly Gaussian.
- The script used in the simulation is available in https://github.com/mjokivuolle/Jokivuolle_MedPhys_2024.

The estimated noise level for the TDD contrast for each patient are documented in Tables S5 and S6. The mean noise levels over the patients were 0.71% ± 0.10% in WM, and 0.43% ± 0.15% in the GTV for both b-values.

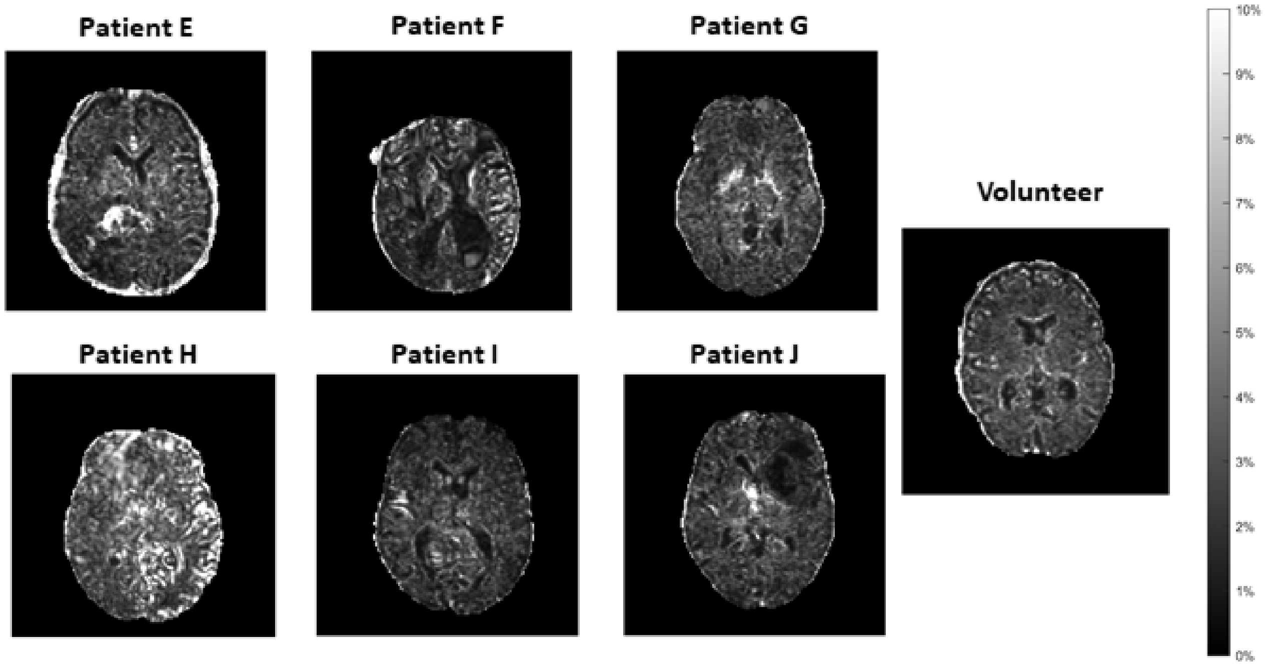

Figure S6. Normalized noise maps of the six patients and the volunteer. The maps show the standard deviation of the repeated b = 0 s/mm^2 scans ($N_{repetitions}$ = 10 for the patients and $N_{repetitions}$ = 20 for the volunteer) divided by the mean. The maps were masked with a brain mask obtained using FMRIB's Brain Extraction Tool. [3,7]

Table S3. Mean signals within the WM ROIs for the noise estimation. The signal values are given in arbitrary units (AU). The two left-most columns show which b-value acquisition is in question, and which diffusion gradient waveform (WF_1 or WF_2) was in use.

		**WHITE MATTER**						
		**Volunteer**	**Patient E**	**Patient F**	**Patient G**	**Patient H**	**Patient I**	**Patient J**
***b*-value (s/mm^2)**	**Gradient waveform**	**Mean signal (AU)**						
**0**	WF_1	2184.03	3360.53	2777.09	2450.52	2530.38	3100.65	2783.38
	WF_2	2200.72	3374.87	2824.04	2538.92	2564.2	3155.73	2807.2
**1250**	WF_1	874.17	2472.04	2128.68	2006.94	2055.16	2376.3	2257.12
	WF_2	881.93	2480.28	2162.74	2050.81	2086.08	2400.86	2278.97
**2500**	WF_1	539.82	788.23	688.83	779.05	814.78	873.8	885.73
	WF_2	556.25	795.06	699.97	801.83	842.92	879.38	906.14

Table S4. Mean signals within the GTVs for the noise estimation. The signal values are given in arbitrary units (AU). The two left-most columns show which *b*-value acquisition is in question, and which diffusion gradient waveform (WF_1 or WF_2) was in use.

		GROSS TUMOR VOLUME					
		Patient E	Patient F	Patient G	Patient H	Patient I	Patient J
b-value (s/mm²)	Gradient waveform	Mean signal (AU)					
0	WF_1	4544.77	3761.17	6792.66	6718.17	4872.98	6901.9
	WF_2	4567.34	3807.04	7025.68	6723.83	4941.27	6883.77
1250	WF_1	3228.36	2836.48	5371.6	4628.57	3866.17	4602.27
	WF_2	3236.89	2880.01	5432.28	4543.47	3898.48	4599.59
2500	WF_1	889.08	792.45	1384.6	862.37	1055.94	797.19
	WF_2	895.78	784.94	1431.8	875.4	1057.02	792.9

Table S5. Estimated noise in simulated TDD_{21} contrast maps for white matter. The left-most column shows for which *b*-value the TDD contrast was simulated.

	WHITE MATTER						
	Volunteer	Patient E	Patient F	Patient G	Patient H	Patient I	Patient J
b-value (s/mm²)	Standard deviation (%)						
1250	0.6899	0.6409	0.7507	0.6734	0.8879	0.6646	0.6343
2500	0.693	0.6419	0.7462	0.673	0.888	0.6666	0.6345

Table S6. Estimated noise in simulated TDD_{21} contrast maps for the GTV. The left-most column shows for which *b*-value the TDD contrast was simulated.

	GROSS TUMOR VOLUME					
	Patient E	Patient F	Patient G	Patient H	Patient I	Patient J
b-value (s/mm²)	Standard deviation (%)					
1250	0.6289	0.5651	0.2482	0.4991	0.3264	0.3399
2500	0.6269	0.5648	0.2473	0.502	0.327	0.3404

The estimated levels of thermal noise in TDD contrast maps were very low. This can be due to the averaging of the DW-MRI signals over the diffusion gradient directions ($N_{directions}$ = 6) and repeated excitations ($N_{excitations}$ = 6 in the simulations, corresponding to the lower limit used in the patient acquisitions), which effectively lowers the thermal noise as $1/\sqrt{N}$, where N is the number of averaged signals. The TDD contrast maps were created from these averaged DW-MRI signals. The noise estimation performed here considered only the thermal noise, while other noise sources, like motion and physiological noise can also contribute to the measurement (see Discussion).

6 Comparison between waveforms in patients

TDD_{21} and TDD_{31} were visually compared in patients C and D (Figure S7). The comparison showed that the overall appearance of the two maps was similar. The large edematous regions in patient D (white arrows) showed no time-dependent contrast in either of the maps, and clear regions of restriction was seen in both maps in and around the GTVs (black arrows). This indicates that that the TDD contrast depends on a change in Δ rather than in δ. The small discrepancies seen in patient D could be due to registration errors.

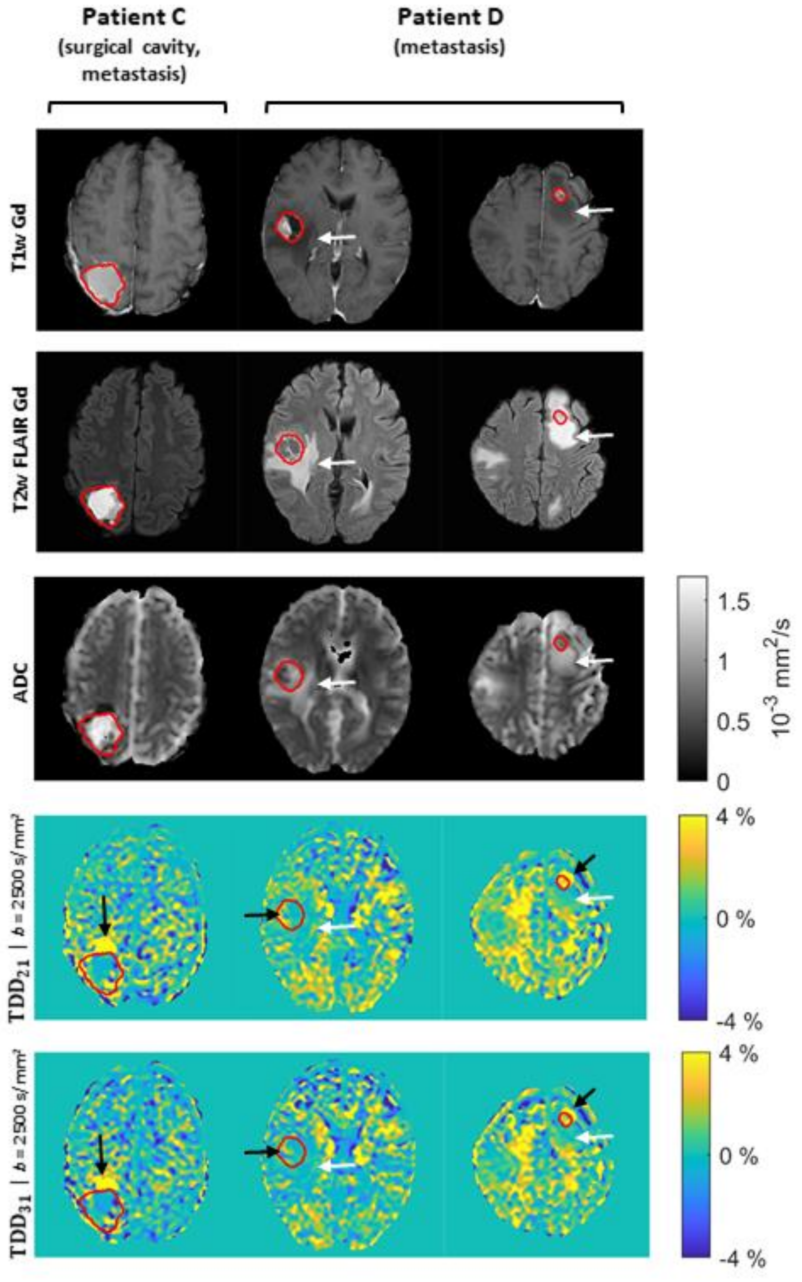

Figure S7. TDD contrasts TDD_{21} and TDD_{31} for patients C and D together with T1-weighted gadolinium contrast image (T1w Gd), T2-weighted FLAIR gadolinium contrast image (T2w FLAIR Gd), and an ADC map. The GTVs are shown in red. White arrows show the large regions of edema in patient D, and black arrows highlight areas of high positive TDD contrast indicating restricted diffusion in and around the GTVs.

References

1. Edelstein WA, Glover GH, Hardy CJ, Redington RW. The intrinsic signal-to-noise ratio in NMR imaging. *Magn Reson Med*. Aug 1986;3(4):604-18. doi:10.1002/mrm.1910030413
2. Dietrich O, Raya JG, Reeder SB, Reiser MF, Schoenberg SO. Measurement of signal-to-noise ratios in MR images: influence of multichannel coils, parallel imaging, and reconstruction filters. *J Magn Reson Imaging*. Aug 2007;26(2):375-85. doi:10.1002/jmri.20969
3. Smith SM, Jenkinson M, Woolrich MW, et al. Advances in functional and structural MR image analysis and implementation as FSL. *Neuroimage*. 2004;23:S208-S219.
4. Zhang Y, Brady M, Smith S. Segmentation of brain MR images through a hidden Markov random field model and the expectation-maximization algorithm. *IEEE Trans Med Imaging*. Jan 2001;20(1):45-57. doi:10.1109/42.906424
5. Jenkinson M, Smith S. A global optimisation method for robust affine registration of brain images. *Med Image Anal*. Jun 2001;5(2):143-56. doi:10.1016/s1361-8415(01)00036-6
6. Jenkinson M, Bannister P, Brady M, Smith S. Improved optimization for the robust and accurate linear registration and motion correction of brain images. *Neuroimage*. Oct 2002;17(2):825-41. doi:10.1016/s1053-8119(02)91132-8
7. Smith SM. Fast robust automated brain extraction. *Hum Brain Mapp*. Nov 2002;17(3):143-55. doi:10.1002/hbm.10062
